# The siderophore transporter Sit1 is involved in the uptake of caspofungin by *Candida albicans*

**DOI:** 10.1128/aac.01236-25

**Published:** 2026-01-29

**Authors:** Andreia Pedras, Catarina Amaral, Cristiano Conceição, Cláudia Malta-Luís, Teresa Pissarro, Carolina V. Mariano, Oscar A. Lenis-Rojas, M. Rita Ventura, Catarina Pimentel

**Affiliations:** 1 Instituto de Tecnologia Química e Biológica António Xavier, Universidade Nova de Lisboa 98819 , Oeiras, Portugal; University of Iowa, Iowa City, Iowa, USA

**Keywords:** yeast, antifungals, iron, echinocandins, siderophore

## Abstract

Invasive infections caused by the yeast *Candida albicans* are a significant health concern due to their high rates of mortality and morbidity. Current treatment guidelines recommend echinocandin drugs as a primary therapeutic option. Despite the prevailing view that these drugs act extracellularly, evidence suggests that the echinocandin caspofungin can enter fungal cells via an as-yet-unidentified mechanism, with the implications of this internalization remaining unknown. Based on our previous findings demonstrating that caspofungin can bind iron and considering its structural resemblance to ferrichrome-type cyclic hexapeptide siderophores, we hypothesize that siderophore transporters could be involved in caspofungin uptake. In *C. albicans*, at the transcriptional level, caspofungin mimics a high iron response, which correlates with the increase in intracellular iron levels in drug-treated cells. This increase depends on the siderophore transporter Sit1, as a *C. albicans* mutant lacking Sit1 does not experience such an increase. Heterologous expression of Sit1 made *Saccharomyces cerevisiae* cells more sensitive to caspofungin, while *C. albicans* cells lacking Sit1 or a *S. cerevisiae* mutant of all siderophore transporters were more tolerant to the drug. Experiments using a newly synthesized fluorescent caspofungin molecule and mass spectrometry confirmed that caspofungin accumulation in yeast is partially mediated by Sit1. Overall, this work identifies siderophore transporters as important players in caspofungin uptake and tolerance in yeast.

## INTRODUCTION

The dimorphic yeast *Candida albicans*, which naturally inhabits the gastrointestinal tract and the vagina, is a leading cause of nosocomial bloodstream infections (1). Iron availability is essential for *C. albicans* colonization, and its acquisition during infection is considered critical (2–5). The commensal and pathogenic lifestyles of *C. albicans* have led to the emergence of mechanisms that enable rapid adaptation to environments where iron is either scarce or overly abundant (2, 6).

Iron is tightly restricted within the host due to the presence of iron-binding proteins, such as transferrin and ferritin, which sequester iron and limit its availability to pathogens (7). As a result, *C. albicans* has evolved a wide range of mechanisms to acquire iron from the host, including a high-affinity reductive system, ferritin and transferrin utilization, a hemoglobin–iron uptake system, and the siderophore transporter Sit1 for the uptake of xenosiderophores (2). In iron-depleted environments, the transcription factor Sef1 activates the expression of genes involved in iron uptake, while Hap43, which is induced by Sef1, represses genes associated with iron-consuming processes and further contributes to the activation of iron uptake genes (6, 8). In contrast, in the gastrointestinal tract, where iron is abundant, the transcriptional repressor Sfu1 inhibits Sef1, thus repressing iron import (4, 6, 9). Thus, Sef1 is considered a promoter of virulence, allowing *C. albicans* to survive in iron-deficient environments, while Sfu1 sustains commensalism by protecting *C. albicans* from iron toxicity in the gastrointestinal tract (6, 10).

Besides playing a pivotal role in *C. albicans* survival and proliferation across different niches, iron has been shown to affect its susceptibility to antifungal drugs through multiple mechanisms. For instance, iron deprivation enhances membrane fluidity, leading to increased passive diffusion of drugs and consequently greater susceptibility (11). Conversely, high iron levels induce changes in the cell wall, rendering cells more resistant to drugs that specifically target this structure (12, 13). Furthermore, genes encoding putative drug exporters in *C. albicans* are differentially expressed depending on iron availability (4). We have also recently reported that iron can directly bind to the echinocandin caspofungin, reducing its efficacy (14).

Echinocandins are the recommended antifungals for treating life-threatening infections caused by *Candida* spp., also known as invasive candidiasis, due to their fungicidal activity, low toxicity, and broad-spectrum activity (15). These drugs inhibit the activity of β-1,3-D-glucan synthase, a transmembrane protein complex responsible for the synthesis of the cell wall component β-1,3-D-glucan, ultimately leading to cell lysis (16). Echinocandins act by binding non-competitively to the catalytic subunit of β-1,3-D-glucan synthase, Fks1 (17), but the binding mechanism is not yet fully understood. It is speculated that they do not need to enter the cell to exert their antifungal activity, and that their hydrophobic tail may intercalate into the plasma membrane (18). However, the existence of an unidentified transporter mediating the uptake of caspofungin has been put forward (19). Consistent with this, recent studies have shown that caspofungin can accumulate in the vacuoles of *C. albicans* clinical isolates, with higher accumulation correlating with increased resistance to caspofungin (20, 21).

The ability of caspofungin to bind iron (14), along with its structural resemblance to cyclic hexapeptide hydroxamate siderophores—typically used by *C. albicans* as a source of iron via import through Sit1 (22, 23)—prompted us to investigate the putative involvement of this transporter in the uptake of caspofungin. Our findings clearly indicate that Sit1 is important for the cellular uptake of caspofungin and contributes to the drug’s antifungal activity.

## MATERIALS AND METHODS

### Strains, growth conditions, and plasmids

Yeast strains used in this study are listed in Table S1. Strains were maintained in YPD agar plates. Unless otherwise stated, all yeast cultures were grown to exponential phase at 30°C in SC medium at pH 6.5 composed of 0.77 g/L Complete Supplement Mixture (MP Biomedicals), 1.7 g/L yeast nitrogen base w/o amino acids and ammonium sulfate, and 5.4 g/L ammonium sulfate, 2% glucose. Whenever the SC medium was supplemented with FeSO_4_, 5 mM citrate buffer was included in its preparation. This concentration of citrate buffer was selected because this is the lowest concentration that allows the use of a wide range of FeSO_4_ concentrations (up to 100 mM) without causing iron precipitation (Fig. S1A). For survival assays, *C. albicans* cultures were left untreated or treated with 0.375 µg/mL caspofungin for 3 h, and serial dilutions of treated or untreated cultures were plated on YPD agar plates for colony-forming units (CFUs) count. Because at 37°C, *C*. *albicans* produces hyphae, and cells tend to aggregate, normalization based on OD_600_ or CFU counts becomes difficult and unreliable. Therefore, all assays with *C. albicans* in liquid medium were performed at 30°C and, for the sake of consistency, assays on solid medium were also conducted at the same temperature. Gradient diffusion tests (E-tests) were performed according to the CLSI standard M44. *C. albicans* inoculum was prepared by suspending colonies grown on YPD plates in phosphate-buffered saline (PBS). Cell density was adjusted with PBS to obtain a stock suspension 1.44 × 10^6^ cells/mL, corresponding to a 0.5 McFarland density standard. Cell suspensions were evenly spread onto SC agar plates, and caspofungin minimal inhibitory concentration (MIC) Test Strips (Liofilchem) were placed on the center of the plate using sterile tweezers. Plates were incubated at 30°C, and growth was evaluated after 24 h. The double-mutant *Sc*Δ*fks1ccc1* was generated using the microhomology PCR method (24) with the oligonucleotides: CCC1_KO_H_F: 5′ atgtccattgtagcactaaagaacgcagtggtgacccATCGTACGCTGCAGG 3′ and CCC1_KO_H_R: 5′ ttaacccagtaacttaacaaagaaccaagccgcacctTTAGGGAGACCGGCAGAT 3′. Successful deletion of *CCC1* from the ScΔ*fks* background was assessed by PCR analysis of genomic DNA using specific oligonucleotides for the *CCC1* gene (pCCC1-261: 5′ GTGACCCGCATTTTTTGTTTTC 3′/CCC1-A4: 5′ TTTTCTATTGCCAAGAGGCC). To construct the *SIT1* expressing plasmid, p*PGK-SIT1*, the *CaSIT1* ORF (C2_08050C_A) was amplified by PCR with the proof-reading polymerase Phusion (Thermo Scientific) using specific oligonucleotides (CaSIT1_Fwd: 5′ ATGACATCTTACCAGTCTTCCAATAATC 3′/BamHI_CaSIT1_Rv: 5′ CGGGATCCCTAAACAGCTACTCTTTTCTTCTTGAAATTG 3′). The PCR product was digested with BamHI, inserted into the SmaI-BamHI digested p*PGK* plasmid (25). The HA sequence was inserted into the p*PGK-SIT1* plasmid (p*PGK-SIT1*-HA) by site-directed mutagenesis using the oligonucleotides SIT1_HA_Rv:- 5′ AGCGTAATCTGGAACATCGTATGGGTAAACAGCTACTCTTTTCTTCTTG 3′ and SIT1_HA_Fwd: 5′ TACCCATACGATGTTCCAGATTACGCTTAGGGATCCATTGAATTGAATTG 3′). All constructs were confirmed by sequencing before downstream use. For *S. cerevisiae* assays using strains transformed with the p*PGK-SIT1* plasmid or the corresponding empty vector, SC medium lacking uracil (prepared as indicated above, using Complete Supplement Mixture without uracil, MP Biomedicals) was supplemented with 0.003% adenine hemisulfate when strain YPH499 was used. Spot assays were carried out by spotting 5 µL of sequential dilutions (from 8 × 10^6^ to 80 cells/mL for *C. albicans* and 1 × 10^6^ to 2 × 10^3^ cells/mL for *S. cerevisiae*) of exponentially growing cultures onto SC agar plates containing the indicated treatments. The plates were incubated at 30°C. Spot assays were repeated at least twice.

### Immunoblot assay

Cells transformed with the plasmid p*PGK-SIT1*-HA were grown to late exponential phase and treated with 5 mM FeSO_4_, 0.375 µg/mL caspofungin, or a combination of both for 2 h. Total proteins were extracted using lysis buffer (50 mM HEPES, pH 7.5, 1 mM EDTA, 100 mM KCl, 10% glycerol, 0.1% NP-40) supplemented with Complete EDTA-free (Roche) and 1 mM PMSF. A total of 100 µg of protein was resolved on a 12% SDS-PAGE gel and transferred to a nitrocellulose membrane. The HA-tagged version of Sit1 was detected using an anti-HA–Peroxidase high-affinity antibody (3F10, Roche). Signals were visualized with SuperSignal West Pico (Thermo Scientific). As a loading control, Pgk1 levels were assessed using an anti-Pgk1 antibody (Life Technologies), followed by incubation with a secondary anti-mouse IgG antibody conjugated to Alexa Fluor 488 (Thermo Scientific). Images were captured using the iBright FL1500 Imaging System (Thermo Scientific).

### RNA sequencing

*C. albicans* SC5314 cultures were left untreated or treated with 5 mM of FeSO_4_, 0.375 µg/mL caspofungin, or a combination of both for 2 h. At this concentration, iron does not precipitate in SC citrate medium and does not affect the growth of *C. albicans* SC5314 (Fig. S1B). Cells were harvested, and RNA was isolated using the phenol:chloroform method (26). RNA samples were then treated with DNase and purified using the RNeasy Kit (Qiagen). Library preparation and sequencing of RNA samples were performed at the Genomics facility of Instituto Gulbenkian da Ciência, Portugal, using the SMART-SEQ2 protocol, adapted from reference (27), and an Illumina NextSeq500 platform (single end, 75-bp read length, 20 M reads), respectively. The quality of the RNA sequencing (RNA-Seq) raw data was analyzed using the FastQC tool. Reads were mapped against the *C. albicans* genome (GCA_000182965.3 downloaded from the NCBI genome database) using Bowtie2 (28), resulting in more than 80% of the reads being aligned. The mapping files were sorted by genomic position using Samtools (29), and the quantification of the transcripts’ expression was done using the featureCounts program (30). Differential expression analyses were done with the R package edgeR (31). We considered all transcripts with a False Discovery Rate (FDR) correction of the *P*-value lower than 0.05 significant, and we further filtered our results by establishing cut-offs of expression (log_2_CPM – counts per million – higher than 3) and fold-change (FC – higher than 2). Enrichment analysis was performed with PANTHER (32), using GO biological process annotation, Fisher’s exact test type, and Bonferroni correction for multiple testing.

### Iron measurements

Cultures were left untreated or treated overnight with 5 mM FeSO_4_, 0.375 µg/mL caspofungin, or a combination of both. Cells were harvested and washed with 10 mM EDTA and metal-free water. Total iron intracellular content was measured by inductively coupled plasma atomic emission spectroscopy (ICP-AES) at REQUIMTE–LAQV, Universidade Nova de Lisboa, Caparica, Portugal. Data were normalized against OD_600_. All assays were performed in biological quadruplicates.

### Chemical synthesis of fluorescent caspofungin

#### General methods

NMR spectra including one-dimensional and two-dimensional (2D) were recorded on Bruker Avance 400 or 500 MHz spectrometers in MeOH-d_4_ with chemical shift values (δ) in parts per million (ppm) referenced with respect to the MeOH signal (quintet, center line δ = 3.310 ppm) and ^13^C NMR were obtained at 100 MHz and chemical shifts are reported as δ values in (ppm) referenced with respect to the MeOH signal (septet, center line δ = 49.0 ppm). The NMR spectra of all synthesized compounds are provided in the Supporting Information (Fig. S2 to S14). Flash preparative column chromatography has been done with silica gel 60H and analytical TLC with aluminum-backed silica gel 60 F254. Visualization was done using a phosphomolybdic acid stain or with a UV lamp. Reagents and solvents were purified and dried according to the literature, when needed (33). All reactions were carried out under an inert atmosphere (argon), except when the solvents were undried. Molecular mass was determined by ESI-MS, and the mass spectra of the samples were acquired in positive mode using Q Exactive Focus. The reactions were followed by analytical HPLC coupled with a PDA detector, Waters 600E/U6K instrument, using a Nova-Pack C18 4 µm, 3.9 mm × 150 mm reverse phase column. The HPLC method used a flow rate of 1 mL/min. The mobile phase consisted of Solvent A (0.1% TFA in water) and Solvent B (90% acetonitrile). The gradient program was as follows: starting at 95% eluent A and 5% eluent B, linearly increasing to 100% eluent B by 12 min, maintaining 100% eluent B from 12 to 15 min, then decreasing to 95% eluent A by 19 min, and holding at 95% Solvent A until 22 min.

#### Synthetic procedures

##### Compound 2

Caspofungin (diacetate salt, 60.0 mg, 0.0549 mmol) was dissolved in a 1:1 mixture of dioxane and water (0.6 mL). To this solution, di-*tert*-butyl dicarbonate (Boc_2_O) (120.3 mg, 0.55 mmol) was added. The reaction mixture was stirred at room temperature for 48 h, with progress monitored by HPLC-PDA, tracking the decrease of the starting material at 7.89 min and the emergence of a new compound signal at 13.94 min. Once the reaction was complete, the solvent was removed under vacuum. The crude product was then purified by silica gel column chromatography (90:10, CH_2_Cl_2_/CH_3_OH) to yield **2** as a white solid (68.1 mg, 89%). HRESI-MS m/z calculated for C_67_H_112_N_10_O_21_H^+^, 1393.8076; found [M+H]^+^, 1393.7753. 1393.7753. ^1^H NMR (400 MHz, MeOH-d_4_) δ 7.15 (d, *J* = 8.1 Hz, 2H), 6.80–6.73 (m, 2H), 4.93 (s, 1H), 4.62–4.49 (m, 5H), 4.47–4.20 (m, 4H), 4.13–3.70 (m, 7H), 3.29–3.09 (m, 4H), 2.49–2.14 (m, 4H), 2.14–1.87 (m, 4H), 1.73–1.57 (m, 4H), 1.55–1.18 (m, 47H), 1.17–1.01 (m, 3H), 0.97–0.82 (m, 10H). ^13^C NMR (101 MHz, MeOH-d_4_) δ 176.15, 172.84, 158.51, 158.35, 132.97, 129.55, 116.25, 80.08, 79.83, 75.81, 71.16, 70.19, 68.86, 58.56, 57.18, 49.85, 49.39, 46.77, 45.91, 38.05, 36.84, 35.38, 34.72, 32.88, 31.22, 31.11, 30.70, 30.58, 30.32, 28.87, 28.82, 27.99, 26.71, 20.73, 20.19, 11.58.

##### Compound 3

Compound **2** (45.2 mg, 0.032 mmol) was dissolved in DMF (1.6 mL), followed by the addition of cesium carbonate (16.3 mg, 0.50 mmol, 1.5 equivalents). The mixture was stirred in an ice bath for 10 min, and after this, 80 wt.% propargyl bromide solution in toluene (3.7 µL, 0.032 mmol) was added. The ice bath was then removed, and the reaction was allowed to proceed overnight at room temperature. Progress was monitored by LC-MS, tracking the disappearance of the starting material **2** [M-H]^+^, m/z 1393.8076) and the appearance of compound **3** ([M-H]^+^, m/z 1431.8233). Once maximum conversion was achieved, the solvent was evaporated under vacuum. The residue was purified by silica gel column chromatography (80:20, CH_2_Cl_2_/CH_3_OH), yielding **3** as a white solid (31.2 mg, 67%). HRESI-MS m/z calculated for C_70_H_114_N_10_O_21_H^+^, 1431.8233; found [M+H]^+^, 1431.7898. ^1^H NMR (400 MHz, MeOH-d_4_) δ 7.27 (d, *J* = 8.3 Hz, 2H), 6.97 (d, *J* = 8.6 Hz, 2H), 4.94 (s, 1H), 4.72 (d, *J* = 2.4 Hz, 2H), 4.66–4.21 (m, 9H), 4.13–3.70 (m, 7H), 3.30–3.16 (m, 4H), 2.93 (t, *J* = 2.4 Hz, 1H), 2.48–2.13 (m, 4H), 2.07–1.94 (m, 4H), 1.69–1.88 (m, 3H), 1.58–1.18 (m, 48H), 1.17–1.04 (m, 3H), 0.96–0.84 (m, 10H). ^13^C NMR (101 MHz, MeOH-d_4_) 176.16, 172.87, 159.00, 158.58, 158.38, 135.28, 129.43, 124.29, 115.92, 79.80, 76.76, 58.59, 56.63, 49.64, 49.43, 49.21, 49.00, 48.79, 48.57, 48.36, 45.92, 38.06, 36.85, 34.72, 32.90, 31.23, 31.12, 30.71, 30.33, 28.87, 28.82, 28.01, 20.73, 20.19, and 11.58.

##### Compound 4

Compound **3** (6.0 mg, 0.0042 mmol) and fluorescein azide dye (4.0 mg, 0.0087 mmol) were dissolved in DMF (0.5 mL). Catalytic amounts of CuI, sodium ascorbate, and 2,2′-bipyridine were then added to the solution. The reaction mixture was stirred at 60°C for 2 h, and the progress was monitored by ESI-MS, observing the disappearance of **3** ([M+H]^+^ m/z 1431.8233) and the formation of **4** ([M+H]^+^ m/z 1889.9459). After completion, the reaction mixture was diluted with acetonitrile:H_2_O (1:4) and purified on a reverse-phase C18 silica gel column using acetonitrile in H_2_O with 0.1% TFA (vol/vol) and as mobile phase, a gradient from 10% to 90% acetonitrile was used to yield **4** as a green powder (6.1 mg, 77%). HRESI-MS m/z calculated for C_94_H_132_N_10_O_27_H^+^, 1889.9459; found [M+H]^+^, 1889.9487. ^1^H NMR (500 MHz, MeOH-d_4_) δ 8.10 (t, *J* = 4.9 Hz, 2H), 8.04 (s, 1H), 7.63 (d, *J* = 6.4 Hz, 2H), 7.28–7.24 (m, 2H), 6.97 (t, *J* = 9.3 Hz, 2H), 6.70 (s, 3H), 6.57 (dt, *J* = 8.8, 2.3 Hz, 2H), 5.11 (s, 1H), 5.04 (s, 1H) 4.60–4.43 (m, 9H), 4.11–3.72 (m, 4H), 3.72–3.51–3.34 (m, 4H), 3.17 (s, 3H), 2.45–2.12 (m, 11H), 1.62 (s, 3H), 1.47 (s, 10H), 1.42–1.29 (s, 48H), 1.42–1.01 (m, 3H), 0.96–0.80 (m, 10H).^13^C NMR (126 MHz, MeOH-d_4_) 175.30, 173.31, 172.87, 168.49, 159.58, 158.59, 135.33, 130.77, 130.27, 129.59, 125.60, 116.22, 116.00, 103.71, 80.11, 62.82, 58.58, 49.75, 49.63, 49.51, 49.46, 49.37, 49.33, 49.31, 49.30, 49.30, 49.29, 49.28, 49.27, 49.26, 49.17, 49.00, 48.83, 48.66, 48.49, 45.93, 38.36, 38.18, 38.07, 36.85, 33.08, 32.90, 31.24, 31.12, 30.85, 30.78, 30.76, 30.75, 30.74, 30.68, 30.47, 30.41, 28.88, 28.82, 28.01, 24.17, 23.74, 20.73, 20.19, 18.74, 16.69, 14.42, and 11.57.

##### Compound 1

Compound **4** (3.5 mg, 0.019 mmol) was dissolved in isopropanol (0.3 mL) and treated with 37% HCl in H_2_O (0.1 mL). The reaction mixture was stirred at room temperature for 2 h, and the progress was monitored by HPLC-PDA, observing the disappearance of the starting material peak at 14.00 min and the appearance of the product peak (**1**) at 9.39 min. Upon completion, the solvent was removed under vacuum, and the reaction mixture was diluted with acetonitrile H_2_O (1:4). The mixture was then purified on a reverse-phase C18 silica gel column using a mobile phase of acetonitrile in H_2_O with 0.1% TFA (vol/vol), applying a gradient from 10% to 90% acetonitrile. Compound **1** was obtained as a green powder (2.5 mg, 85%). HRESI-MS m/z calculated for C_79_H_108_N_14_O_21_H^+^, 1589.7886; found [M+2H]^2+^, 795.3979. ^1^H NMR (500 MHz, MeOH-d_4_) δ 9.15 (s, 2H), 9.04 (s, 2H), 8.28 (d, *J* = 8.2 Hz, 5H), 7.65–7.58 (m, 2H), 7.52 (d, *J* = 8.8 Hz, 2H), 7.36 (s, 1H), 7.25 (d, *J* = 8.2 Hz, 1H), 7.19 (d, *J* = 4.9 Hz, 2H), 6.53 (d, *J* = 3.1 Hz, 2H), 5.30 (s, 2H), 5.23 (s, 1H), 5.14 (d, *J* = 8.5 Hz, 2H), 5.02–4.97 (m, 2H), 4.63 (s, 3H), 4.41 (d, *J* = 7.0 Hz, 3H), 4.31 (d, *J* = 5.1 Hz, 3H), 4.19 (d, *J* = 7.3 Hz, 2H), 4.06 (q, *J* = 4.2 Hz, 2H), 4.01 (d, *J* = 9.9 Hz, 2H), 3.86 (s, 2H), 3.81–3.73 (m, 3H), 3.35 (s, 8H), 3.07 (s, 4H), 2.38–2.28 (m, 6H), 2.16 (d, *J* = 14.3 Hz, 4H), 2.03 (s, 5H), 1.60 (s, 6H), 1.29 (d, *J* = 3.4 Hz, 27H), 1.16 (s, 3H), 1.14 (s, 3H), 0.93–0.88 (m, 6H). ^13^C NMR (126 MHz, MeOH-d_4_) 176.93, 174.86, 173.37, 172.75, 168.94, 163.37, 162.97, 162.82, 159.80, 130.09, 120.59, 105.22, 98.79, 74.92, 71.32, 68.34, 66.97, 65.86, 64.75, 56.10, 50.33, 49.85, 49.51, 49.34, 49.17, 49.00, 48.83, 48.66, 48.49, 47.17, 45.93, 42.95, 39.13, 38.09, 37.10, 36.86, 34.63, 34.47, 33.07, 32.90, 31.24, 31.17, 30.83, 30.77, 30.74, 30.63, 30.59, 30.46, 30.40, 30.38, 30.33, 28.06, 27.01, 25.24, 24.17, 23.73, 20.71, 20.17, 20.00, 16.68, 14.43, and 11.57.

### Quantification of CAS-FAM

Cultures were left untreated or treated with 1 µM CAS-FAM for 1 h. Cell cultures (50 mL) were harvested, washed, and resuspended in 1 mL of dH_2_O. ODs were normalized to the lowest OD. Cells were lysed with glass beads in a bead beater (two cycles of 1 min at 6 m/s). The crude homogenate was centrifuged at 20,000 × *g* for 10 min at 4°C. The supernatant was centrifuged at 100,000 *g* for 30 min, and 200 µL was transferred to a black NUNC microplate for fluorescence measurement (excitation: 484/20; emission: 530/25).

### Quantification of caspofungin by liquid chromatography-mass spectrometry

Sample preparation for liquid chromatography-mass spectrometry (LC-MS) was performed as described in reference (34) with modifications. Cultures were left untreated or treated with 1 µg/mL caspofungin for 2 h. Cell cultures (100 mL) were harvested, washed with milli-Q H_2_O, and resuspended in 500 µL of LC-MS grade H_2_O. ODs were normalized to the lowest OD. Acetonitrile (200 µL) was added, and cells were lysed with glass beads in a bead beater (1 min at 6 m/s). The homogenate was centrifuged at 20,000 × *g* for 10 min at 4°C. 200 µL of mobile phase A (20 mM of ammonium acetate and 1% acetic acid in water) was added to the supernatants. Samples were analyzed at UniMS–ITQB NOVA, Oeiras, Portugal. The analysis was carried out using a Dionex Ultimate 3000 UHPLC coupled to QExactive Orbitrap Focus, Thermo. An electrospray ionization source was used. The UHPLC was performed using water with 20 mM of ammonium acetate and 1% acetic acid in water as mobile phase A and 1% acetic acid in acetonitrile as mobile phase B. The 56.87-min gradient program was as follows: 0–28.37 min, 25%–70% B; 28.37–28.87 min, 70%–98% B; 28.87–46.47 min, 98% B; 46.47–46.87 min, 98%–25% B; 46.87–56.87 min, 25% B. The column temperature was maintained at 30°C. A flow rate of 400 μL/min was used at 0–28.87 min and 51.87–56.87 min, and a flow rate of 500 μL/min was used at 28.87–51.87 min. Data were acquired with the ddMS2 method, which consisted of several cycles of Full MS scans (*R* = 70,000; scan range = 93.4–1,400 m/z) followed by 3 ddMS2 scans (R = 17,500; 20, 40, 60 NCE) in positive mode. Data were analyzed with the Compound Discoverer 3.2 (Thermo) software. Identification of compounds was performed at two levels; Level 2b: identification by accurate mass (with an accepted deviation of 3 ppm), and MS/MS spectra; Level 3: identification by accurate mass alone (with an accepted deviation of 3 ppm).

### Microscopy

Cultures were left untreated or treated with 1.5 µM CAS-FAM for 1.5 h. Cell cultures were harvested by centrifugation, washed, and resuspended in PBS. Z-series stacks were acquired using an Axio Observer Z1 microscope controlled with the ZEISS Zen (Blue Edition) software. Imaging was acquired with a 63× Plan-Apochromat 1.4NA DIC oil immersion objective (ZEISS), a light source LED Module of 475 nm, with an excitation wavelength range of 450–488 nm and emission wavelength range of 501–527 nm, and Bright Field. Fluorescence intensities of stained cells (*n* ≥ 100) were quantified using Fiji (35).

## RESULTS

### Caspofungin interferes with *Candida albicans* iron homeostasis

We have recently shown that caspofungin binds iron (14). To assess whether this effect could also impact yeast iron homeostasis, RNA-Seq was performed to analyze the transcriptional landscape of *C. albicans* cells exposed to iron (Fe), caspofungin (CAS), or a combination of both compounds (Fe + CAS). Differential gene expression analyses (relative to untreated cells) were conducted using edgeR (31), and applying a set of restrictive parameters (log_2_FC > 1, log_2_CPM > 3, and FDR < 0.05, Tables S2 to S4) to identify the most significantly affected biological processes.

**Fig 1:**
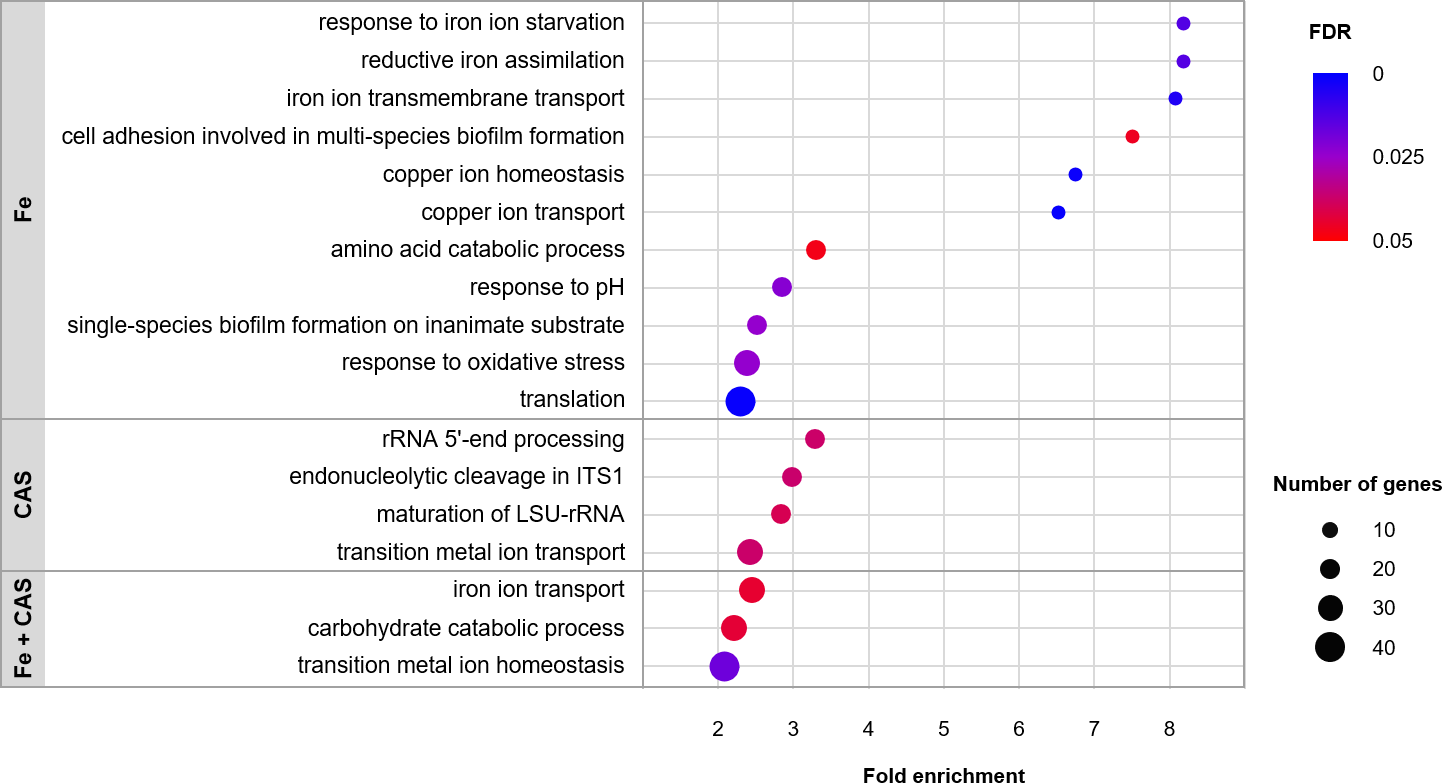
Functional enrichment of *C. albicans* gene expression after treatment with iron, caspofungin, or both. Enrichment analysis of the differentially expressed genes. Only enriched categories (FDR < 0.05 and fold enrichment > 2) are represented. The size and color of the circles represent the number of genes associated with each term and FDR, respectively.

Genes whose expression was affected by the individual or combined treatment conditions are listed in Tables S2 to S4. The functional categorization of the differentially expressed genes is represented in Fig. 1. Genes with altered expression under Fe treatment were grouped into 11 enriched categories associated with iron and copper homeostasis, adhesion, biofilm formation, filamentation, response to pH and oxidative stress, and translation. The differentially expressed genes in CAS treatment fall into three enriched categories related to rRNA processing and one category of transition metal ion transport. In Fe + CAS treatment, three categories of genes were enriched: iron ion transport, carbohydrate catabolic process, and cellular transition metal ion homeostasis (Fig. 1).

In *C. albicans*, the adherence of yeast cells and the growth of hyphae, or filamentation, are essential steps in biofilm formation, which involve changes in the cell wall (36). Therefore, genes that code for proteins involved in biofilm formation and cell wall synthesis or remodeling are interconnected and often have overlapping roles. These genes are known to be repressed under high iron conditions (4); accordingly, under Fe treatment, we observed a downregulation of genes encoding proteins required for adhesion (*ALS1*, *ALS2*, *ALS3*, *ALS7*, *HYR3*, *MSB2*, *PGA7*, *PGA10*, *PSA2*), filamentation (*AMS1*, *CSA1*, *RBT5*), and cell wall remodeling, including chitin synthase *CHS2* and glycosidases for the cross-linking of β-1,3- and β-1,6-D-glucan (*PHR1*, *PHR3*) (36–38) (Table S2). *CSA1* and *RBT5* genes are also involved in hemoglobin utilization (39), further contributing to their downregulation under this condition. However, four adhesins, including *ALS1*, were upregulated in CAS treatment, in agreement with a study demonstrating that caspofungin causes cell aggregation mediated by Als1 (40). Caspofungin also induced the expression of chitin synthases (*CHS2*, *CHS3*, *CHS7*) (Table S3), consistent with the compensatory synthesis of chitin triggered by caspofungin inhibition of β-1,3-D-glucan synthesis, through the activation of calcineurin, PKC, and HOG signaling pathways (37). Genes encoding mannosyltransferases (*MNT2*, *KTR4*, *PMT4*, *PMT5*, *PMT6*) and β-1,3-D-glucan remodeling and cross-linking (*ROT1*, *CRH11*, *EXG2*, *PHR1*, *PHR2*) were also upregulated, likely to counteract the cell wall alterations imposed by caspofungin.

Biofilm regulation involves a highly complex transcriptional network of more than 50 transcriptional regulators controlled by a core of nine master regulators (36, 41–43). We found four of these master regulators to be differentially expressed under the tested conditions, with *BRG1*, *EFG1*, and *TEC1* being repressed in Fe treatment, and *BCR2* upregulated in CAS and Fe + CAS treatments.

The cellular response to pH, which is enriched in Fe treatment, is mediated by the pH-responsive transcription factor Rim101 that has been shown to positively regulate filamentation and iron acquisition in neutral and alkaline environments (44–46). Consistently, we observed a repression of *RIM101* in Fe treatment (Table S2). The “cellular response to oxidative stress” category is also enriched under this condition and comprises genes involved in oxidative stress detoxification, such as the catalase *CAT1* and superoxide dismutase *SOD1* that were induced in response to Fe and Fe + CAS treatments (Tables S2 and S4), possibly to mitigate the oxidative damage imposed by iron, which can generate ROS via Fenton-type reaction (4). *CAT1* was also upregulated, along with *SOD5,* following caspofungin exposure (Table S3), corroborating the caspofungin-induced production of ROS, previously reported (47).

**Fig 2:**
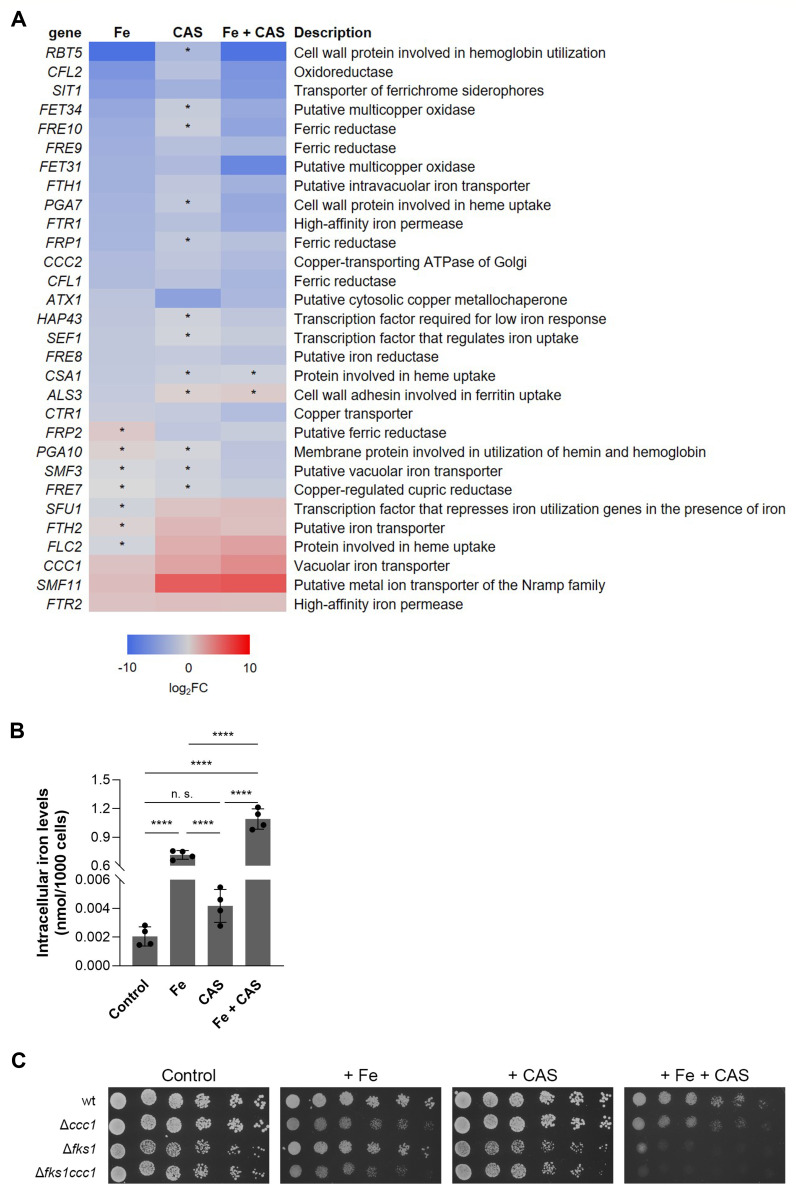
Caspofungin affects iron homeostasis. (**A**) Heatmap depicting *C. albicans* genes related to the subcategory of iron homeostasis, which were differentially expressed under Fe, CAS, or Fe + CAS conditions. For each condition, the log_2_ fold change (log_2_FC) of the selected transcripts is indicated using a color code. Genes significantly upregulated (log_2_FC > 1) or downregulated (log_2_FC < –1) in response to Fe, CAS, or Fe + CAS (treated versus untreated conditions) are shaded in red or blue, respectively. Asterisks (*) mark genes differentially expressed with FDR < 0.3 and/or log_2_CPM > 1.3 and therefore do not meet the more stringent criteria of log₂FC > 1, log_2_CPM > 3, and FDR < 0.05 and are not listed in Tables S2 to S4. The complete data set is available at NCBI GEO under accession number GSE280500. *S. cerevisiae* gene names were used whenever a *C. albicans* gene name was not assigned. (**B**) The iron content of *C. albicans* SC5314 cells left untreated (Control) or treated overnight with Fe, CAS, or both (Fe + CAS) was determined by ICP-AES. Significance of differences was calculated using one-way ANOVA with Tukey’s HSD *post hoc* test (****P < 0.0001; n.s. not significant). (**C**) Growth of *S. cerevisiae* wild type (wt, BY4742), Δ*ccc1*, Δ*fks1,* and Δ*fks1ccc1* strains on SC agar plates (Control) containing Fe (5 mM), CAS (0.03 µg/mL), or Fe + CAS, for 3 days at 30°C.

Remarkably, all treatments, including caspofungin alone, led to a significant enrichment of processes related to metal homeostasis (Fig. 1), with genes involved in iron homeostasis being particularly well represented (Fig. 2A). In *C. albicans*, iron acquisition via the reductive system involves the reduction of ferric iron to the ferrous form by ferric reductases (48–50), with the subsequent reconversion of the ferrous iron to the ferric form by multicopper oxidases (51), followed by its transport through the iron permease, Ftr1, which forms a complex with the multicopper oxidases (52–54). In agreement, genes encoding ferric reductases (*CFL1*, *CFL2*, *FRE9*, *FRE10*, *FRP1*, *FRE8*), multicopper oxidases (*FET31*, *FET34*), *FTR1*, and the putative vacuolar iron permease (*FTH1*) involved in iron release into the cytosol (55) were downregulated in Fe and Fe + CAS treatments, except the iron permease *FTR2*, whose expression is regulated in an opposite manner (54). Since multicopper oxidases require copper to catalyze the oxidation of iron, this metal is essential for iron homeostasis. Copper is imported by the high-affinity transporter Ctr1 and then scavenged by the metallochaperone Atx1, which delivers copper to the copper ATPase Ccc2 located in the post-Golgi compartment and required for the insertion of copper in multicopper oxidases (56–58). The genes encoding these proteins (*CTR1*, *ATX1*, *CCC2*) were downregulated in the three conditions. Other mechanisms of iron acquisition include (i) the siderophore transporter Sit1, which enables the uptake of xenosiderophores (22, 23), (ii) the adhesin Als3, involved in iron utilization from ferritin (59), and (iii) a system for hemoglobin/heme utilization (39), whose respective genes (*SIT1*, *ALS3,* and *RBT5*, *PGA7,* and *CSA*) were also downregulated. The transcription factors responsible for low-iron response (*HAP43*, *SEF1*) (6, 8) were similarly downregulated.

Many iron uptake genes were downregulated in CAS treatment, while five genes were upregulated (Fig. 2A): *SMF11*, a putative transporter required for the uptake of manganese (Mn), which is needed to activate Mn-dependent metalloenzymes, such as Mn-SODs (60); *CCC1*, a vacuolar iron transporter for storage and detoxification in *S. cerevisiae*, known to be induced in response to high iron levels (61); *FLC2*, involved in heme uptake (62); *FTH2*, a putative high-affinity iron transporter for intravacuolar iron storage (55); and the transcription factor *SFU1* that regulates the cell response to high iron (6, 9). Overall, caspofungin alone appears to elicit a transcriptional response similar to that of Fe treatment. Interestingly, the typical high iron gene expression profile (shut down of genes involved in iron uptake and upregulation of those responsible for iron sequestration in the vacuoles [6]) is more pronounced in Fe + CAS treatment, in terms of fold change, than in Fe treatment alone.

The striking observation that caspofungin, at the transcription level, partially mimicked an iron overload response led us to investigate whether the drug could impact intracellular iron accumulation. As such, we assessed the total iron content of *C. albicans* cells treated with Fe, CAS, and Fe + CAS. Corroborating the observed gene expression profile, iron levels were significantly higher in Fe + CAS treatment compared to iron alone, and caspofungin treatment resulted in a modest increase in iron compared to the control, although this latter difference did not reach statistical significance (Fig. 2B). To further explore the link between intracellular iron accumulation and caspofungin, we used a *Saccharomyces cerevisiae* Δ*fks1* mutant to minimize the impact of caspofungin extracellular activity. Unlike *C. albicans* (63), this yeast remains viable without *FKS1* due to the presence of a homolog, *FKS2*, which has partially overlapping functions (64). Because the *CCC1* gene encodes a vacuolar iron transporter that plays a key role in maintaining metal homeostasis by sequestering excess iron into the vacuole (61), we constructed the double-mutant *Sc*Δ*fks1ccc1* and assessed its sensitivity, and that of the corresponding single mutants, to Fe, CAS, and Fe + CAS (Fig. 2C). Interestingly, the *Sc*Δ*fks1* mutant showed pronounced sensitivity to Fe + CAS, and this effect was even greater in the *Sc*Δ*fks1ccc1* double mutant. These findings further support a functional connection between iron and caspofungin that appears to be independent of the drug’s extracellular activity.

### The *C. albicans* siderophore transporter Sit1 affects yeast sensitivity to caspofungin

**Fig 3:**
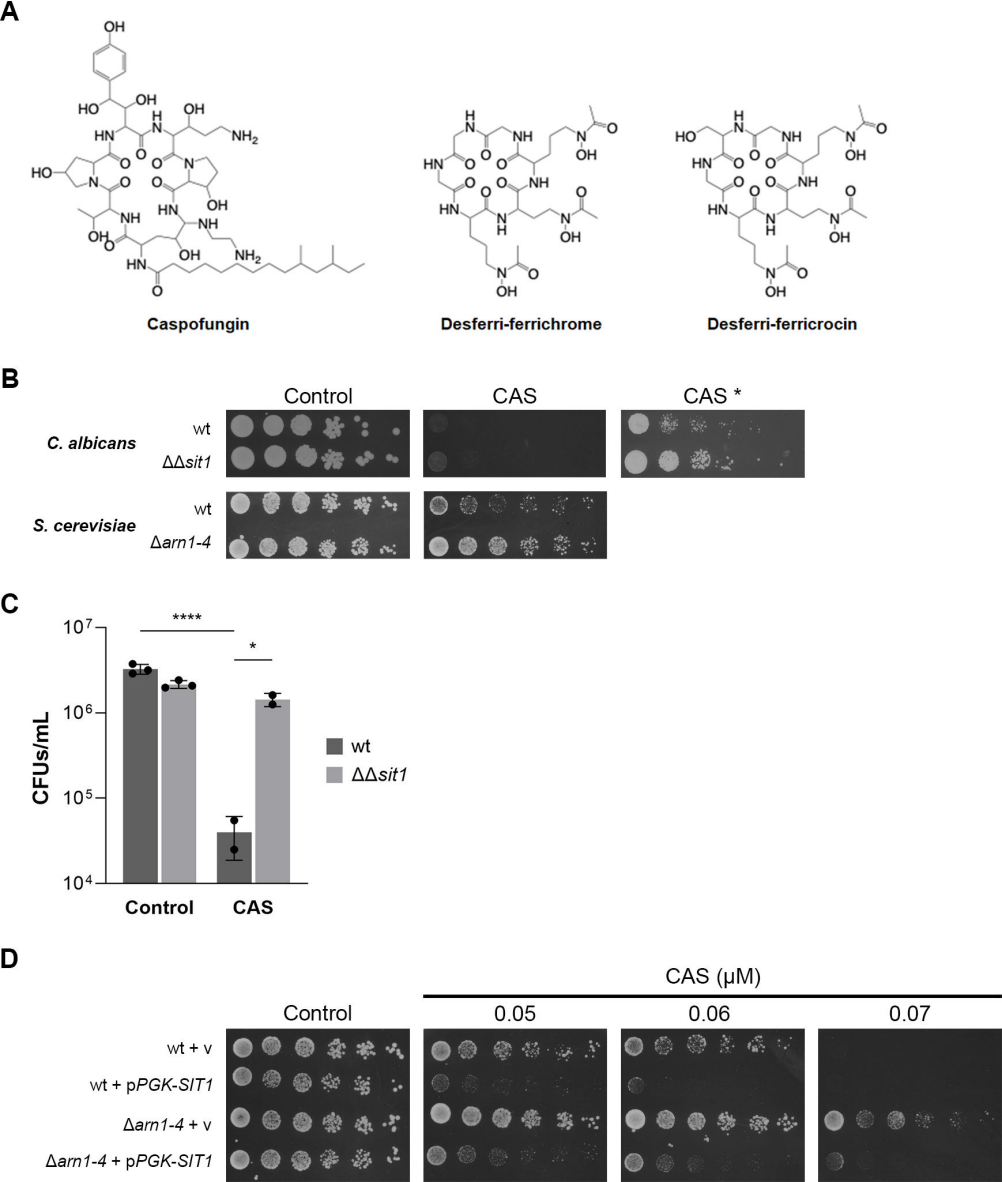
Sit1 affects caspofungin efficacy against yeast. (**A**) Structural similarity between caspofungin and cyclic hexapeptides hydroxamate siderophores. (**B**) Growth of *C. albicans* wt (CAF2-1) and ΔΔ*sit1* mutant strains on SC agar plates (Control) containing 0.4 µM caspofungin (CAS), for 48 h or 5 days (CAS *) at 30°C. Growth of *S. cerevisiae* wt (YPH499) and Δ*arn1-4* mutant strains on SC agar plates (Control) containing 0.1 µM caspofungin (CAS), for 48 h at 30°C. (**C**) *C. albicans* cells were left untreated (Control) or treated with 0.375 µg/mL caspofungin (CAS) for 3 h and plated on YPD agar plates for CFU count. Significance of differences was calculated using two-way ANOVA with Tukey’s HSD *post hoc* test (***P < 0.001; *P < 0.05). (**D**) Growth of *S. cerevisiae* wt (YPH499) and Δ*arn1-4* mutant strains transformed with a plasmid containing the *CaSIT1* gene under the control of *ScPGK1* promoter (p*PGK-SIT1*) or with the empty vector (v) on SC-ura agar plates (Control) containing the indicated concentrations of caspofungin (CAS), for 48 h at 30°C.

The Chemical Entities of Biological Interest, a dictionary of molecular entities focused on small chemical compounds (65), revealed that caspofungin shares structural similarities with several siderophores (Fig. 3A), including ferrichrome and ferricrocin (similarity ≥ 25%). The three molecules possess a macrocyclic core, obtained from cyclization of a peptide backbone, a core present in many natural siderophores and antifungal agents. Siderophores like ferrichrome and ferricrocin have hydroxamate functional groups, which are responsible for their strong iron(III) affinity (66). Caspofungin does not present the same groups but does feature multiple hydroxyamino acid residues and amines, contributing to the molecule’s polarity and the ability to interact with metals (14). Ferrichrome-like siderophores can be imported by *C. albicans* through the siderophore transporter Sit1 as a source of iron (22, 23, 67). *SIT1* is one of the most downregulated genes in response to caspofungin treatment (Fig. 2A; Table S3). These findings led us to evaluate the relevance of Sit1 in enabling yeast to withstand caspofungin stress. As shown in Fig. 3B and C, *C. albicans* ΔΔ*sit1* cells exhibited significantly reduced sensitivity to caspofungin and other echinocandins (Fig. S15) compared to the wild-type strain. Consistently, reintroduction of *SIT1* into the ΔΔ*sit1* mutant strain increases its sensitivity to caspofungin (Fig. S16). Although the caspofungin MIC remains unchanged in the mutant compared to the wild-type strain (Fig. S17), it is nevertheless clear from both solid (Fig. 3B) and liquid media assays (Fig. S18) that the absence of *SIT1* alleviates the negative impact of the drug on *C. albicans* growth.

The effect was also tested in *S. cerevisiae*, which differs from *C. albicans* in that it possesses four siderophore transporters: Arn1, which is the most similar to *C. albicans* Sit1 (*Ca*Sit1), takes up ferrichrome and related hydroxamate-type siderophores (68); Arn2/Taf1 transports triacetylfusarinine C and fusigen (69, 70); Arn3/Sit1 mediates the uptake of ferrioxamine and ferrichrome (71, 72); and Arn4/Enb1 imports enterobactin (73). We observed that a *S. cerevisiae* strain deleted for all four siderophore transporters (Δ*arn1-4*) was more tolerant to caspofungin than the parent strain (Fig. 3B). Reinforcing the role of Sit1 in caspofungin activity, *S. cerevisiae* wild-type and Δ*arn1-4* strains expressing a functional *C. albicans SIT1* (Fig. S19) expressed under the control of the *PGK1* promoter (p*PGK-SIT1*) were strongly sensitive to caspofungin (Fig. 3D).

Overall, these results suggest that siderophore transporters play a significant role in the antifungal activity of caspofungin against yeast.

### Sit1 enhances the uptake of a fluorescent caspofungin derivative by *S. cerevisiae*

**Fig 4:**
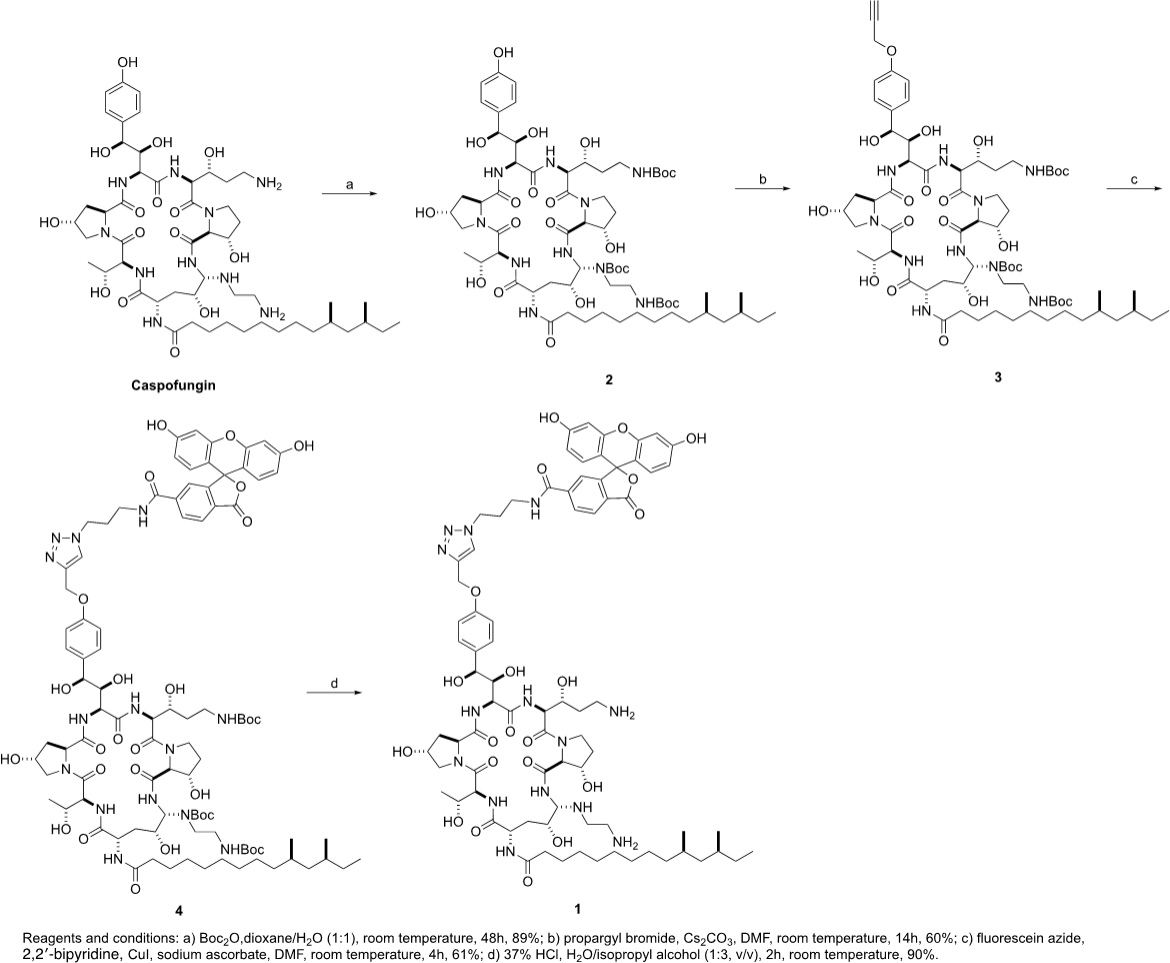
Synthesis of fluorescent FAM-labeled caspofungin (CAS-FAM) (1). Reagents and conditions: (a) Boc_2_O, dioxane/H_2_O (1:1), room temperature, 48 h, 89%; (b) propargyl bromide, Cs_2_CO_3_, DMF, room temperature, 14 h, 67%; (c) fluorescein azide, 2,2′-bipyridine, CuI, sodium ascorbate, DMF, room temperature, 4 h, 77%; (d) 37% HCl, H_2_O/isopropyl alcohol (1:3, vol/vol), 2 h, room temperature, 85%.

As a first approach to determine whether Sit1 could be involved in the uptake of caspofungin by *C. albicans*, a new fluorescein (6-FAM)-labeled caspofungin molecule (CAS-FAM **1**) was designed and synthesized (Fig. 4). Since caspofungin’s ability to bind iron depends on its ethylenediamine moiety (14), we ensured that this group remained free by linking FAM to caspofungin through its phenolic hydroxy group (Fig. 4) (20). Caspofungin has been previously labeled with fluorescein but at the ethylenediamine position by formation of a thiourea with fluorescein isothiocyanate (74). Our synthesis started with the protection of the amine groups with Boc_2_O to afford N-Boc protected caspofungin **2** in 89% yield, followed by selective O-alkylation at the phenol group with propargyl bromide in the presence of cesium carbonate to afford compound **3** in 67% yield, as described by Jaber et al. (20). In fact, we followed the procedure described by these authors to successfully obtain the propargyl functionalized unprotected caspofungin, by removing the N-Boc protecting groups from **3** with HCl, before attempting the click reaction with fluorescein azide. However, in our hands, this reaction was unsuccessful under all the conditions attempted. Thus, we decided to perform the click coupling reaction with protected intermediate **3**, but the corresponding triazole adduct **4** was not obtained. Facing these results, we hypothesized that caspofungin was sequestering the Cu ions necessary for the Cu(I)-catalyzed azide-alkyne cycloaddition to occur. To confirm this assumption, and to overcome this problem, 2,2′-bipyridine was added to the cycloaddition reaction, and **4** was successfully obtained in 77% yield. Removal of the Boc protecting groups with 35% HCl afforded the final compound **1** in 85% yield. This new caspofungin fluorescent probe was obtained in 4 steps in 39% overall yield.

**Fig 5:**
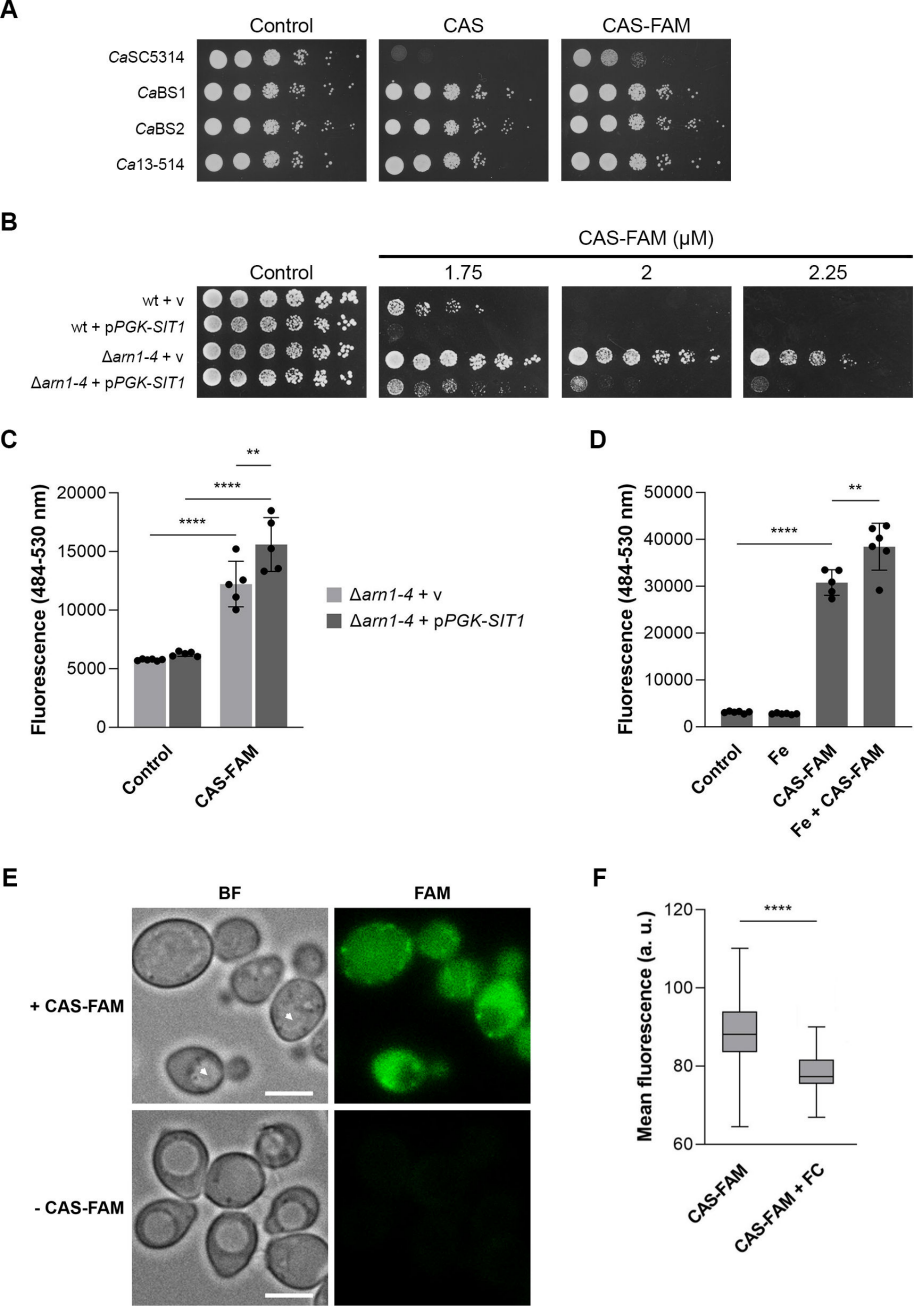
Sit1 mediates the uptake of CAS-FAM. (**A**) Growth of caspofungin-sensitive (*C. albicans* SC5314, laboratory strain) and resistant strains (*Ca*BS1, *Ca*BS2, and *Ca*13-514) on SC agar plates containing 0.4 µM caspofungin (CAS) or 4 µM CAS-FAM, after 24 h at 30°C. (**B**) Growth of *S. cerevisiae* wt (YPH499) and Δ*arn1-4* mutant strains transformed with a plasmid containing the *CaSIT1* gene under the control of *ScPGK1* promoter (p*PGK-SIT1*) or with the empty vector (v) on SC-ura agar plates (Control) containing the indicated concentrations of CAS-FAM, after 48 h at 30°C. (**C**) Quantification of the intracellular fluorescence levels of *S. cerevisiae* cells left untreated (Control) or treated with 1 µM CAS-FAM for 1 h. Significance of differences was calculated using two-way ANOVA with Tukey’s HSD *post hoc* test (****P < 0.0001; **P < 0.01). (**D**) Quantification of the intracellular fluorescence levels of *S. cerevisiae* Δ*arn1-4* cells transformed with p*PGK-*SIT1 left untreated (Control) or treated with 1 µM CAS-FAM (CAS-FAM), 500 µM FeSO_4_ (Fe), or both (Fe + CAS FAM) for 1 h was measured. Significance of differences was calculated using one-way ANOVA with Tukey’s HSD *post hoc* test (****P < 0.0001; **P < 0.01). (**E**) Fluorescence microscopy images of *S. cerevisiae* wt cells transformed with p*PGK-*SIT1 treated with 1.5 µM CAS-FAM (+ CAS FAM) for 1.5 h. Scale bar: 5 μm; arrow heads: vacuoles, BF: bright field. (**F**) Quantification of fluorescence microscopy images of cells treated with 1.5 µM CAS-FAM (CAS-FAM) for 1.5 h, either alone or in combination with 10 µM ferrichrome (CAS-FAM + FC). At least 100 cells were analyzed per condition. Significance of differences was calculated using Student’s T-test (****P < 0.0001).

In terms of antifungal activity, CAS-FAM appeared to be less effective against *C. albicans* than caspofungin, and the subtle but consistent tolerant phenotype of the ΔΔ*sit1* mutant (Fig. 3B) was no longer evident (data not shown). We also performed spot assays using three *C. albicans* strains carrying mutations in different hotspot (HS) regions: BS1 and BS2 (with S645P mutation in HS1) and 13–414 (with R1361G mutation in HS2) (75). As expected, all mutants showed increased tolerance to caspofungin and CAS-FAM compared to the sensitive laboratory strain (*C. albicans* SC5314) (Fig. 5A).

In the case of *S. cerevisiae*, wild-type cells remained more susceptible to CAS-FAM than Δ*arn1-4* cells, and the expression of *C. albicans SIT1* (*CaSIT1*) increased the sensitivity to the drug in both strains (Fig. 5B). Therefore, the Δ*arn1-4* and Δ*arn1-4* expressing *SIT1* strains were used to assess CAS-FAM intracellular accumulation. After a 1-h treatment with CAS-FAM, cell lysates were prepared for fluorescence quantification. As shown in Fig. 5C, CAS-FAM treatment led to increased fluorescence levels in both strains, with a more pronounced increase observed in Δ*arn1-4* cells expressing *CaSIT1*. Given that caspofungin is capable of binding iron (14), the impact of iron on caspofungin uptake was further investigated by repeating the assay with Δ*arn1-4* cells expressing *CaSIT1* with and without iron supplementation. Interestingly, fluorescence levels were higher when cells were exposed to both CAS-FAM and iron compared to CAS-FAM alone (Fig. 5D).

In *S. cerevisiae* wild-type cells expressing *CaSIT1*, CAS-FAM predominantly localizes near the cell surface and in the cytoplasm as distinct bright spots. (Fig. 5E). When *S. cerevisiae* cells were challenged with both CAS-FAM and ferrichrome, a known substrate of *Sc*Arn1/Arn3 and *Ca*Sit1, a sharp decrease in CAS-FAM fluorescence was observed (Fig. 5F).

While these results support the involvement of *Ca*Sit1 in the intracellular uptake of CAS-FAM, they also indicate the existence of additional transporter(s) capable of mediating this process.

### *C. albicans* cells lacking Sit1 accumulate lower amounts of caspofungin under iron-replete conditions

**Fig 6:**
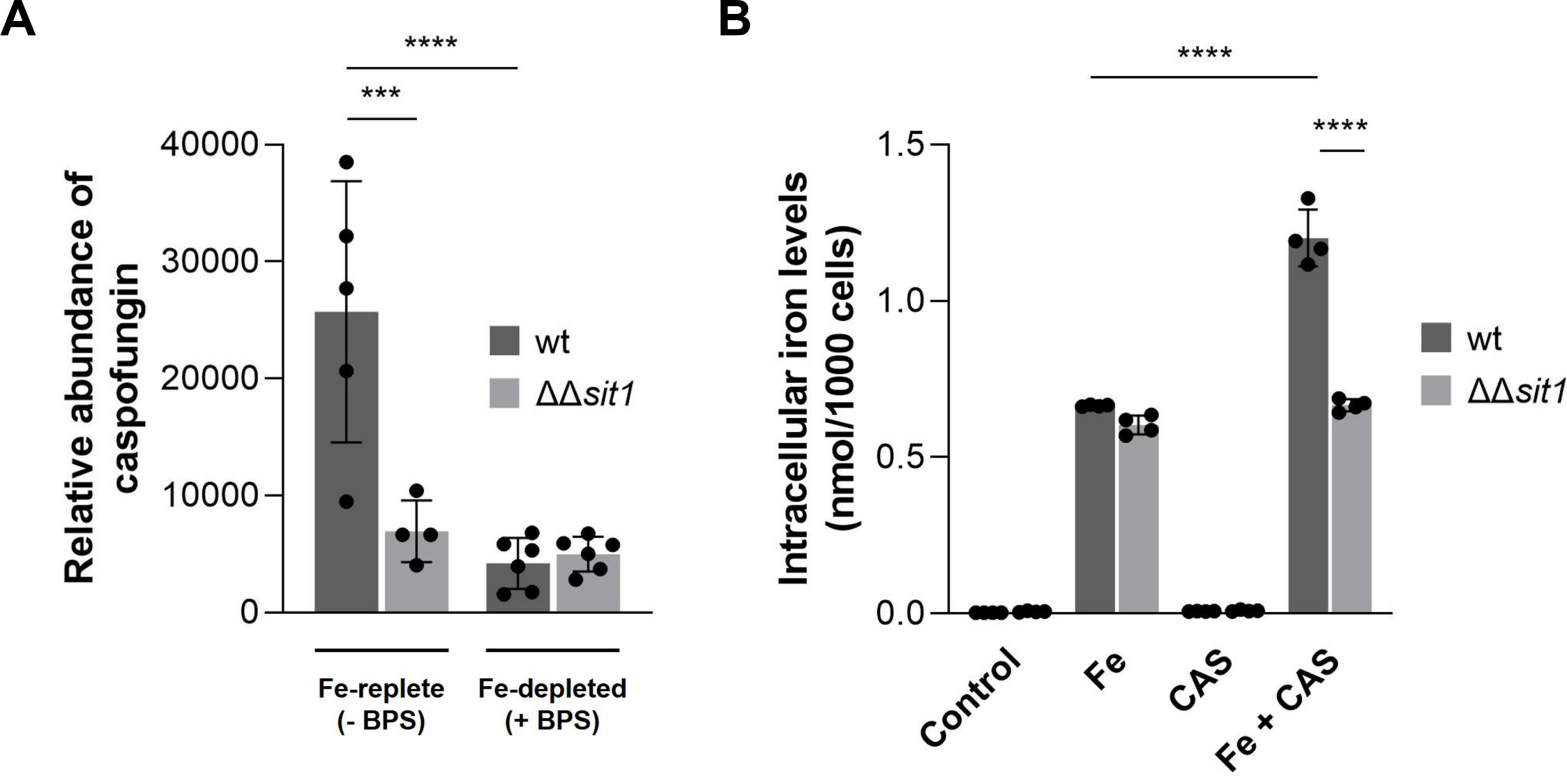
Sit1 is involved in the uptake of caspofungin in *C. albicans*. (**A**) The accumulation of caspofungin in *C. albicans* wt (CAF2-1) and ΔΔ*sit1* cells grown in iron-depleted medium (supplemented with 300 µM of the iron chelator BPS) or iron-replete medium and treated with 1 µg/mL caspofungin for 2 h was measured by LC-MS (****P < 0.0001; ***P < 0.001). (**B**) The iron content of *C. albicans* wt (CAF2-1) and ΔΔ*sit1* cells left untreated (Control) or treated overnight with 5 mM FeSO_4_ (Fe), 0.375 µg/mL caspofungin (CAS), or both (Fe + CAS) was determined by ICP-AES. Significance of differences was calculated using two-way ANOVA with Tukey’s HSD post hoc test (****P < 0.0001).

To confirm the involvement of Sit1 in caspofungin uptake in *C. albicans*, we evaluated caspofungin accumulation in wild-type and ΔΔ*sit1* mutant strains under iron-replete (SC medium containing 0.04 mg/mL iron, as measured by ICP-AES) and iron-depleted conditions (300 µM BPS added) using LC-MS (Fig. 6A). Under iron-replete conditions, the absence of *SIT1* led to lower intracellular caspofungin levels in the mutant compared to the wild-type strain. Under iron-depleted conditions, caspofungin levels remained unchanged in the mutant but dropped significantly in the wild-type, resulting in no detectable difference between the two strains (Fig. 6A).

We also questioned whether the increased intracellular iron levels observed upon caspofungin treatment (Fig. 2B) could be mediated by Sit1. The higher iron levels observed in Fe + CAS conditions compared to Fe alone were no longer evident in the ΔΔ*sit1* mutant (Fig. 6B). These findings support the hypothesis that caspofungin internalization in *C. albicans* depends on Sit1 and iron availability.

## DISCUSSION

While caspofungin is thought to exert its antifungal activity extracellularly, growing evidence suggests that it can enter *C. albicans* cells (19–21, 74). However, how it is taken up and whether its internalization is beneficial or harmful to yeast remains unclear. In this study, we addressed these open biological questions.

Since caspofungin binds iron (14), we hypothesized that an iron transporter might be involved in its uptake. Given that the yeast iron response is regulated at the transcriptional level (4, 6, 8, 9, 46), we assessed the transcriptomic profile of *C. albicans* cells exposed to caspofungin. Interestingly, caspofungin elicited a transcriptional response resembling the high-iron condition, and treated cells exhibited increased iron accumulation (Fig. 2B), supporting our initial hypothesis. *SIT1* was among the most downregulated genes under caspofungin treatment (Fig. 2A). In *C. albicans,* Sit1 is responsible for the transport of ferrichrome-type siderophores, including ferrichrome, ferricrocin, ferrichrysin, ferrirubin, coprogen, and triacetylfusarinine C (23). Although caspofungin lacks the hydroxamate groups, characteristic of ferrichrome-type siderophores, it shares some structural similarities with them (Fig. 3A), and both are synthetized by fungal microorganisms (76–81). These findings prompted us to explore whether Sit1 could be involved in caspofungin transport.

This study provides three lines of evidence supporting the involvement of Sit1 in the cellular uptake of caspofungin. First, using a newly synthesized fluorescently labeled caspofungin, CAS-FAM (Fig. 4), we observed that the heterologous expression of *C. albicans SIT1* in *S. cerevisiae* wild-type and mutant cells deleted for all siderophore transporters (Δ*arn1-4*) resulted in increased intracellular fluorescence levels (Fig. 5C). Second, following caspofungin treatment, the *C. albicans* mutant ΔΔ*sit1* accumulates less drug than the cognate wild-type strain (Fig. 6A). Finally, *C. albicans* wild-type cells accumulate more iron upon exposure to Fe + CAS in a Sit1-dependent manner (Fig. 6B). Interestingly, under iron-depleted conditions, caspofungin levels decreased significantly in the wild-type strain, and the dependence on Sit1 was no longer observed (Fig. 6A). While BPS-induced iron depletion is known to increase *SIT1* expression (67), it likely also sequesters iron from caspofungin, potentially rendering the drug unable to enter the cells, which could explain the reduced accumulation. This result, along with the observation that the Δ*arn1-4* mutant expressing *SIT1* accumulates more caspofungin when grown in iron-supplemented medium (Fig. 5D), strongly supports the hypothesis that caspofungin must bind iron to enter cells.

The potential for siderophore transporters to facilitate the uptake of non-siderophore molecules has been previously reported. In *Aspergillus fumigatus*, *Af*Sit1 has been described to mediate the uptake of VL-2397, a ferrichrome-like antifungal drug with unusual amino acids in its structure (82). Moreover, in bacteria, siderophore transporters have been shown to act as promiscuous transporters of multiple chemically distinct compounds (83, 84).

A previous study has shown that higher accumulation of fluorescent caspofungin derivatives in the vacuole correlated with increased resistance to caspofungin (20, 21). However, our data indicate that caspofungin is harmful when internalized, as *C. albicans* ΔΔ*sit1* is less susceptible to caspofungin than the wild-type strain, despite accumulating lower levels of the drug (Fig. 3B and 6A). Moreover, heterologous expression of *SIT1* in *S. cerevisiae* increases caspofungin accumulation and susceptibility to the drug (Fig. 3D).

Contrary to the previously described fluorescent caspofungin derivatives (20, 21), CAS-FAM does not accumulate in the vacuole; rather, it localizes in speckles near the cell surface and within the cytoplasm (Fig. 5E). Similar observations were made by Sumiyoshi and colleagues while using a caspofungin-FITC derivative (74). Interestingly, CAS-FAM localization is consistent with *Sc*Arn1 and *Ca*Sit1 localization following ferrichrome supplementation (67, 85, 86). Therefore, the different localization of fluorescent caspofungin derivatives may explain why we observed a positive correlation between caspofungin uptake and sensitivity to the drug, while Jaber and colleagues (20) reported the opposite.

The difference in susceptibility to caspofungin between the wild-type and siderophore transporter mutant(s) appears to be more prominent in *S. cerevisiae* than in *C. albicans* (Fig. 3B). Differences between Sit1 and Arn transporters may account for this variation; for instance, the ferrichrome transporter Arn1 from *S. cerevisiae* might have a higher affinity for caspofungin than *C. albicans* Sit1. Another possibility is the existence of an extra still unidentified siderophore transporter in *C. albicans*, as suggested by Heymann and colleagues (23), which could also facilitate caspofungin uptake. Alternatively, some promiscuous transporters unrelated to siderophore transporters may contribute to the yeast uptake of caspofungin. Our data somewhat support this latter hypothesis, as the Δ*arn1-4* mutant is still capable of accumulating CAS-FAM (Fig. 5C).

Remarkably, CAS-FAM is less effective than caspofungin in preventing yeast growth (Fig. 3B and 5B). The larger size of CAS-FAM compared to caspofungin (Fig. 4) may account for this observation, as it might not inhibit Fks1 as efficiently as caspofungin.

In summary, the data presented in this study highlight Sit1 as a key player in caspofungin uptake by yeast cells. Our findings clearly indicate that the intracellular accumulation of caspofungin is detrimental to cell growth, paving the way for future research aimed at uncovering the underlying molecular mechanisms. Moreover, the increased sensitivity to caspofungin in *S. cerevisiae* cells expressing Sit1 suggests that enhancing the (non-vacuolar) intracellular accumulation of the drug could be a strategy to improve its efficacy. This is particularly important in the current context, where resistance to antifungals is on the rise, and the development of new antifungal drugs or new strategies to enhance the efficacy of existing treatments is urgently needed (87, 88).

## Data Availability

All data supporting the findings of this study are available within the paper and its Supplemental material. The data sets presented here can be accessed at https://www.ncbi.nlm.nih.gov/geo/ under accession number GSE280500.

## SUPPLEMENTAL MATERIAL

Supplemental material aac.01236-25-s0001.pdf Table S1; Fig. S1 to S19.

Table S2 aac.01236-25-s0002.xlsx Genes differentially expressed in *C. albicans* cells treated with iron.

Table S3 aac.01236-25-s0003.xlsx Genes differentially expressed in *C. albicans cells treated with caspofungin*.

Table S4 aac.01236-25-s0004.xlsx Genes differentially expressed in *C. albicans cells treated with iron and caspofungin*.

ASM does not own the copyrights to Supplemental Material that may be linked to, or accessed through, an article. The authors have granted ASM a non-exclusive, world-wide license to publish the Supplemental Material files. Please contact the corresponding author directly for reuse.
