# Supplementary material for: The siderophore transporter Sit1 is involved in the uptake of caspofungin by Candida albicans
Source: Antimicrob Agents Chemother. 2026 Jan 29;70(3):e01236-25. doi: 10.1128/aac.01236-25 (PMC12959105; doi:10.1128/aac.01236-25)
Supplement: Supplemental material — Table S1; Fig. S1 to S19. [file aac.01236-25-s0001.pdf]

The siderophore transporter Sit1 is involved in the uptake of caspofungin by *Candida albicans*

[1]Andreia Pedras, [1]Catarina Amaral, [1]Cristiano Conceição, [1]Cláudia Malta-Luís, [1]Teresa Pissarro, [1]Carolina V. Mariano, [1]Oscar A. Lenis-Rojas, [1]M. Rita Ventura, and [1*]Catarina Pimentel

[1] Instituto de Tecnologia Química e Biológica António Xavier, Universidade Nova de Lisboa, Av. República, 2780-157 Oeiras, Portugal.
*corresponding author: pimentel@itqb.unl.pt

Keywords:
Yeast, antifungals, iron, echinocandins, siderophore

Table S1. Yeast strains used in this study.

Strain	Genotype / Description	Source
Candida albicans SC5314	Wild-type strain	ATCC
Candida albicans CAF2-1	*URA3*/*ura3*::*imm434*	[1]
Candida albicans C4-SHB1.1 (ΔΔ*sit1*)	*sit1*Δ::*hisG-URA3-hisG*/*sit1*Δ::*hisG*	[1]
Candida albicans SM1813C (ΔΔ*sit1*[SIT1])	Integration of p1367-CaSITpORF/*Cla*I in a ΔΔ*sit1*	[1]
Candida albicans BS1	Clinical isolate with FKS1 mutations (S645P)	[2]
Candida albicans BS2	Clinical isolate with FKS1 mutations (S645P)	[2]
Candida albicans 13-514	Clinical isolate with FKS1 mutations (R1361G)	[2]
Saccharomyces cerevisiae YPH499	*MAT*a *ura3-52 lys2-801 ade2-101 trp1-63Δ his3-200Δ leu2-1Δ*	[3]
Saccharomyces cerevisiae Δ*arn1-4*	*MAT*a *ura3-52 lys2-801 ade2-101 trp1-63Δ his3-200Δ leu2-1Δ arn1Δ*:*HISG arn2Δ*:*HISG arn3Δ*:*HISG arn4Δ*:*HISG-URA3-HISG*	[3]
Saccharomyces cerevisiae BY4742	*MATα, his3Δ1 leu2Δ0 met15Δ0 ura3Δ0*	EUROSCARF
Saccharomyces cerevisiae BY4742 Δccc1	*MATα, his3Δ1 leu2Δ0 met15Δ0 ura3Δ0 YLR220w::kanMX4*	EUROSCARF
Saccharomyces cerevisiae BY4742 Δfks1	*MATα, his3Δ1 leu2Δ0 met15Δ0 ura3Δ0 YLR342w::kanMX4*	EUROSCARF
Saccharomyces cerevisiae BY4742 Δfksccc1	*MATα, his3Δ1 leu2Δ0 met15Δ0 ura3Δ0 YLR220w::HIS3MX6, YLR342w::kanMX4*	This work

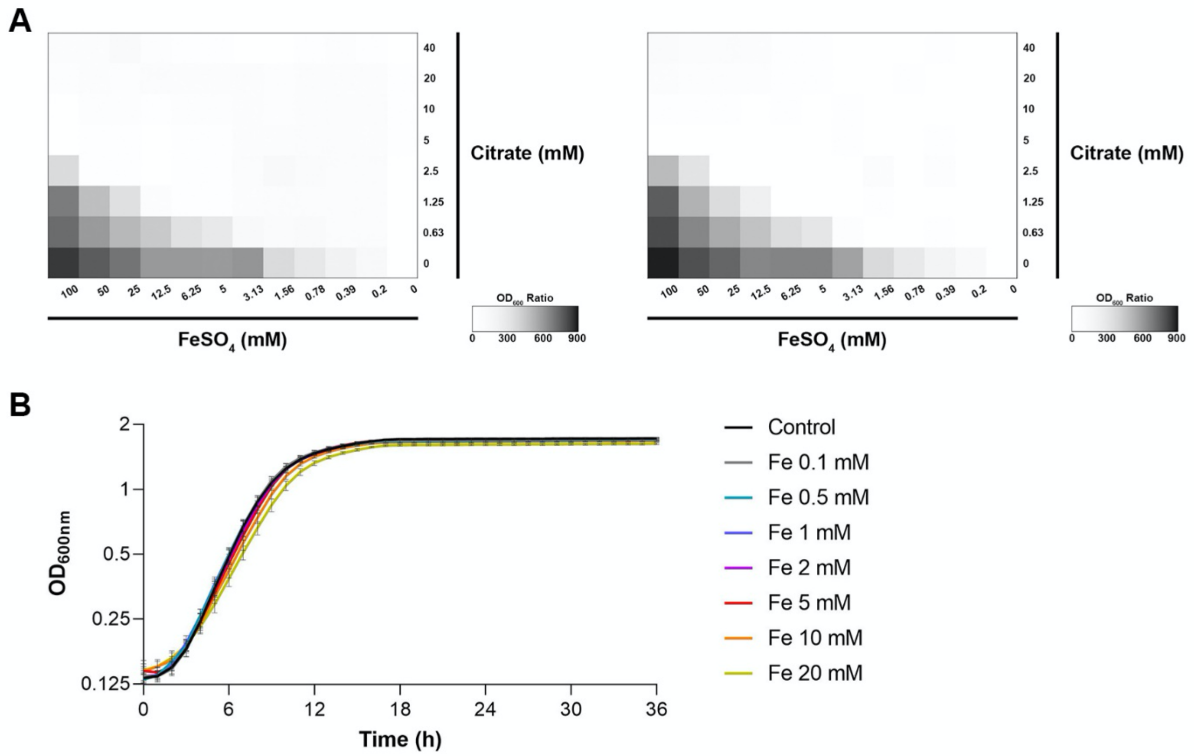

Figure S1. Impact of citrate on $FeSO_4$ precipitation and *C. albicans* growth across a wide range of $FeSO_4$ concentrations (A) A citrate concentration of 5 mM or higher prevents iron precipitation across a wide range of $FeSO_4$ concentrations. SC medium at pH 6.5 was supplemented with the indicated concentrations of citrate (prepared by serial dilutions from a 100 mM citrate buffer, pH 5.5) and combined with increasing concentrations of $FeSO_4$ in a 96-well plate (each well containing a different combination). After incubation at 30 °C for 24 or 48 hours, OD_{600} was measured. Iron precipitation was indicated by increased OD_{600} in the respective wells compared to control conditions (no added iron). **(B)** *C. albicans* growth is not affected by $FeSO_4$ concentrations ranging from 0.1 to 20 mM in the presence of 5 mM citrate buffer. *C. albicans* cells were grown in SC medium buffered with 5 mM citrate at 30 °C for 36 hours in the presence or absence of the indicated iron concentrations. Growth was recorded at 1 hour intervals. Error bars represent mean ± standard deviation.

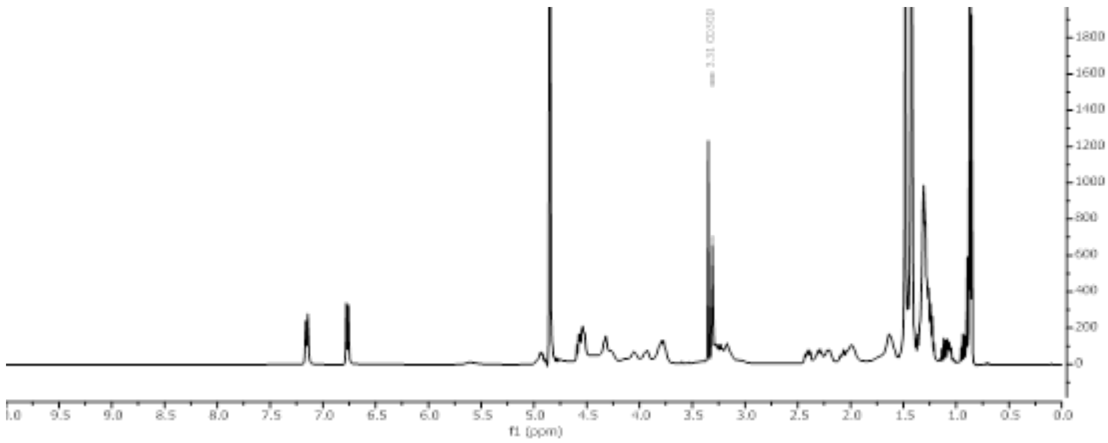

Figure S2. 400 MHz ^{1}H-NMR spectrum of compound 2 in MeOH-d_4.

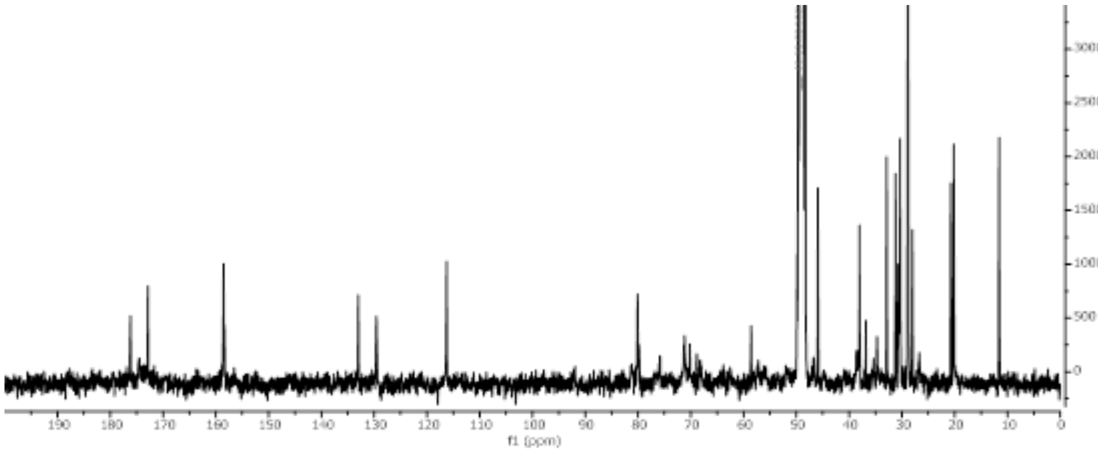

Figure S3. 101 MHz ^{13}C-NMR spectrum of compound 2 in MeOH-d_4.

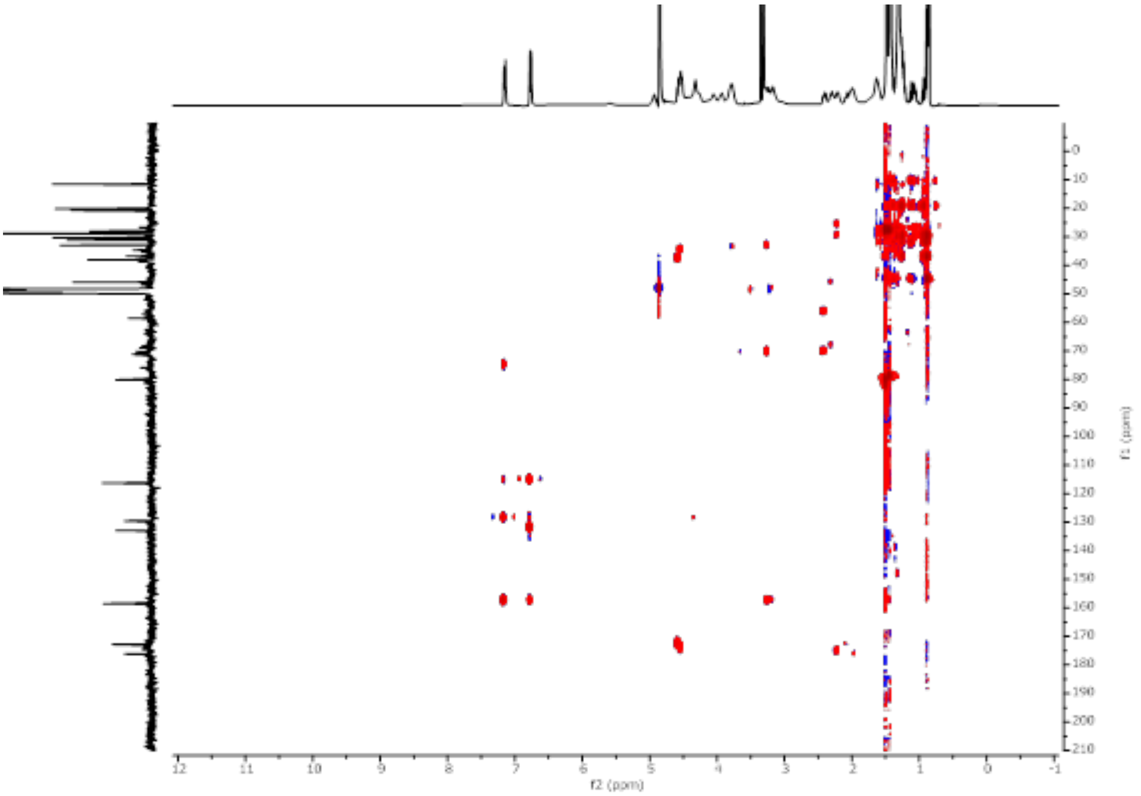

Figure S4. HMBC spectrum of compound 2 in MeOH-d₄.

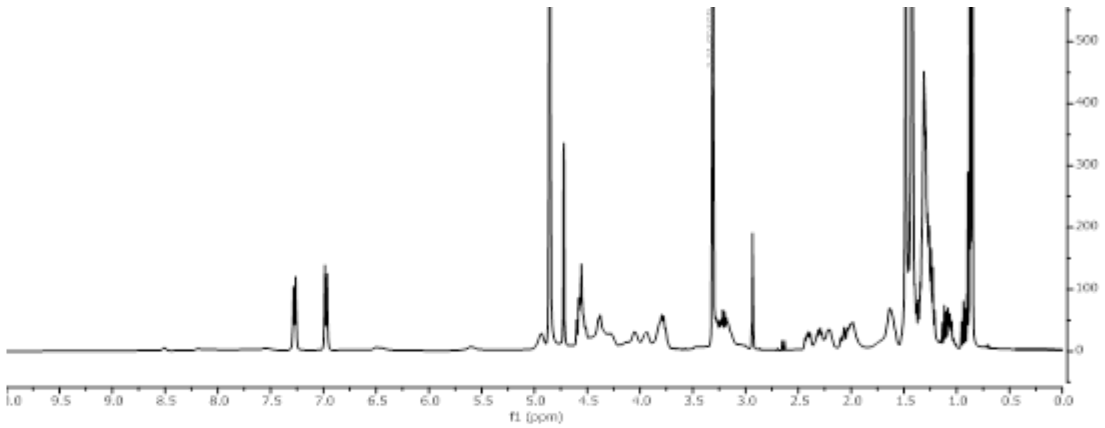

Figure S5. 400 MHz ^{1}H-NMR spectrum of compound 3 in MeOH-d_4.

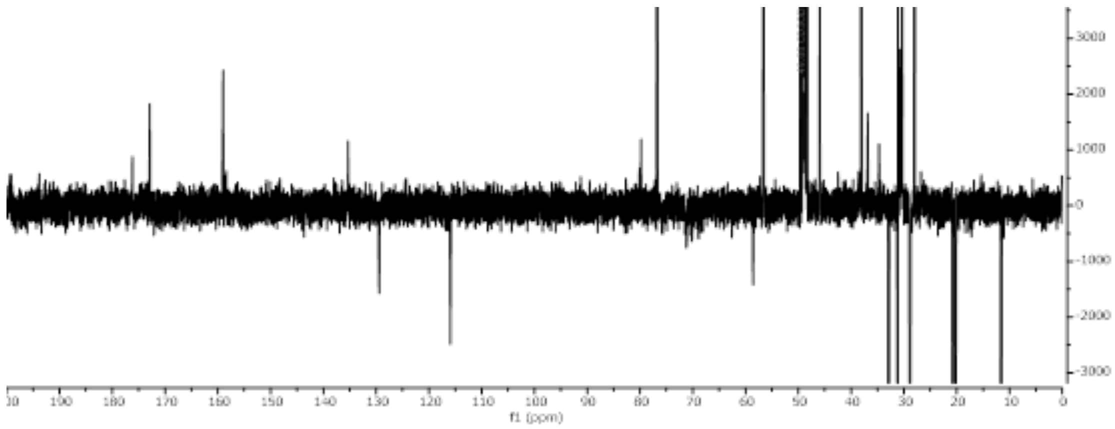

Figure S6. 101 MHz ^{13}APT-NMR spectrum of compound 3 in MeOH-d_4.

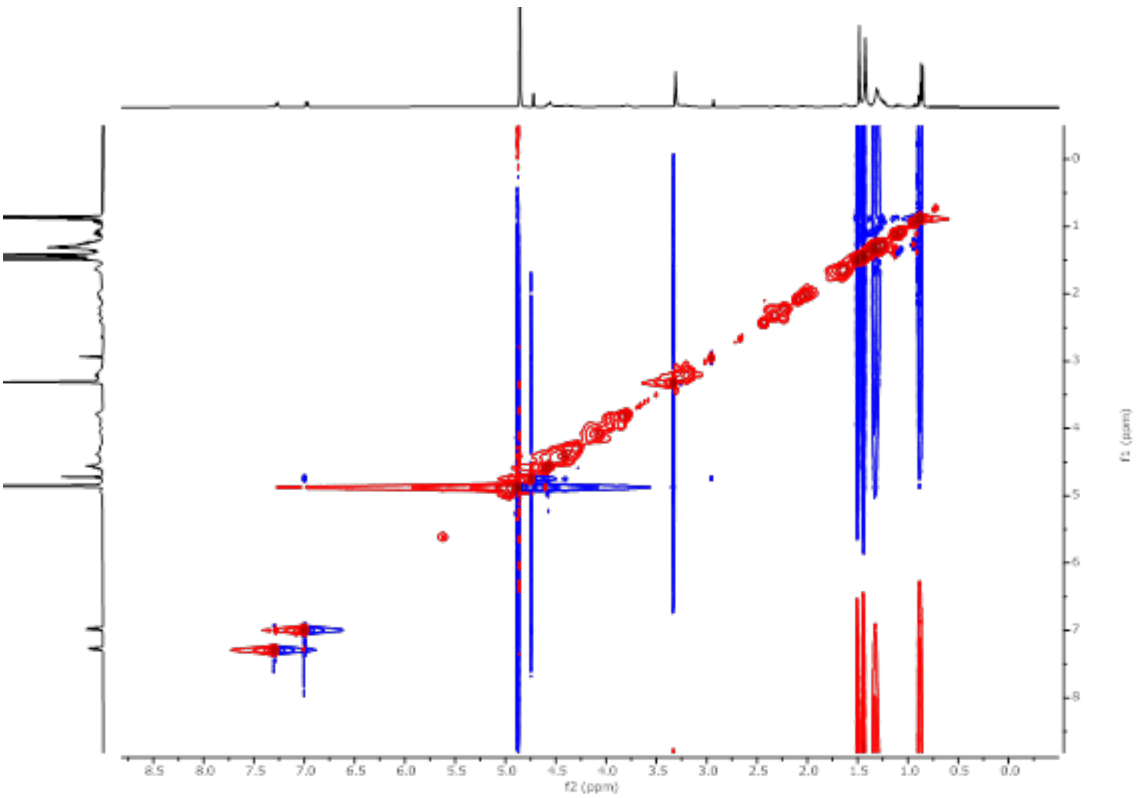

Figure S7. NOESY spectrum of compound 3 in MeOH-d_4.

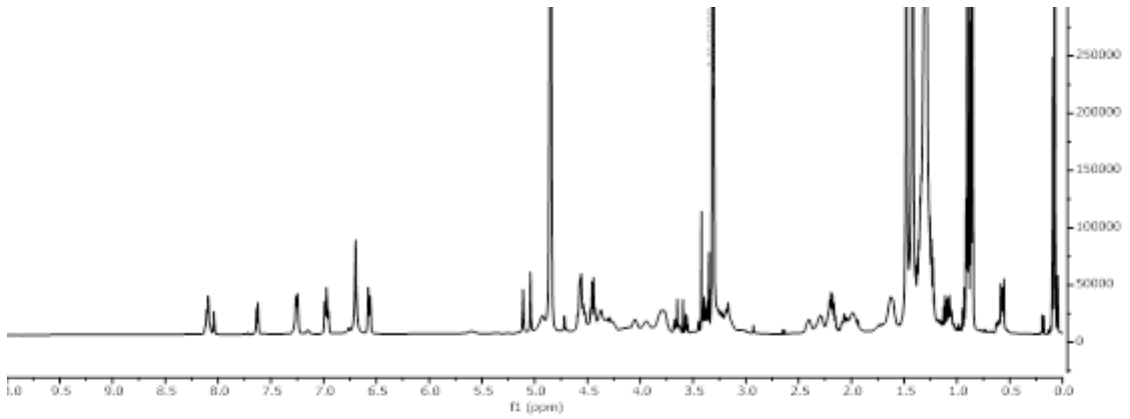

Figure S8. 500 MHz ^{1}H-NMR spectrum of compound 4 in MeOH-d_4.

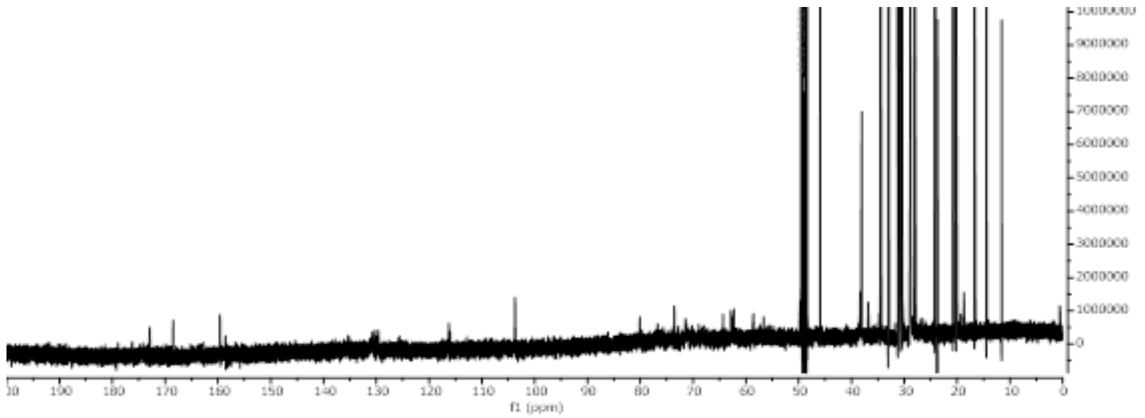

Figure S9. 126 MHz ^{13}C-NMR spectrum of compound 4 in MeOH-d_4.

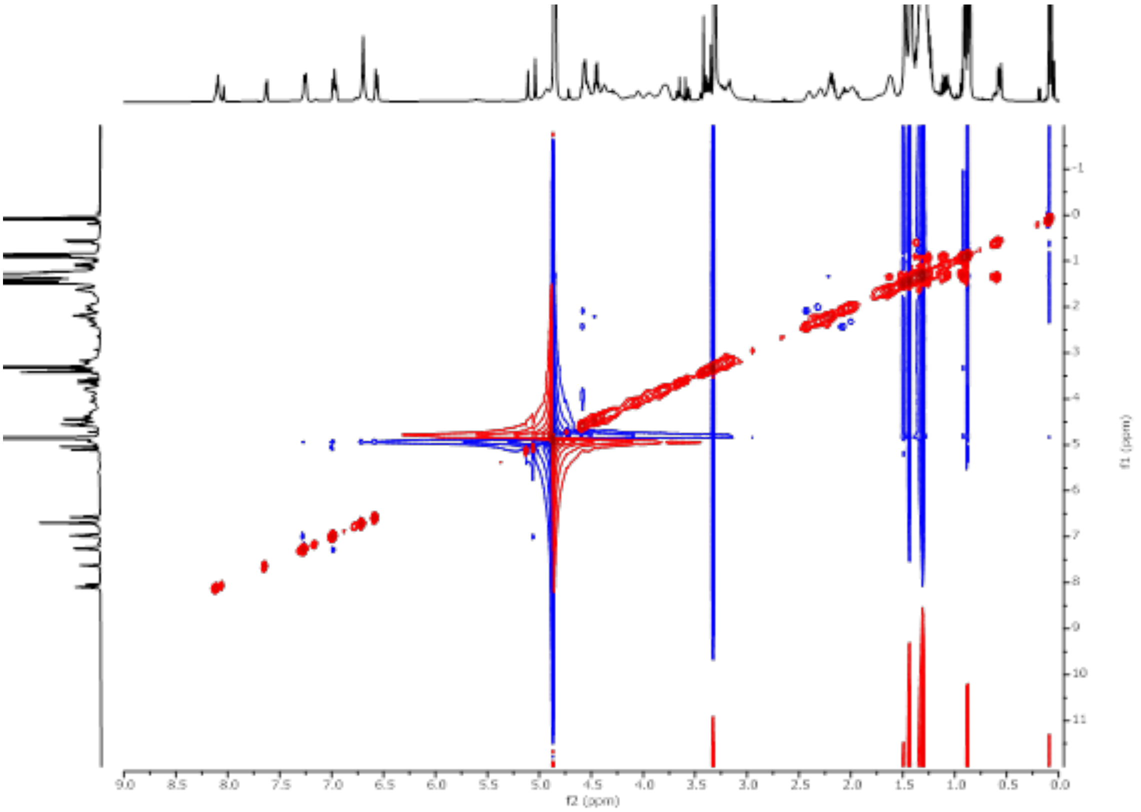

Figure S10. ROESY NMR spectrum of compound 4 in MeOH-d$_4$.

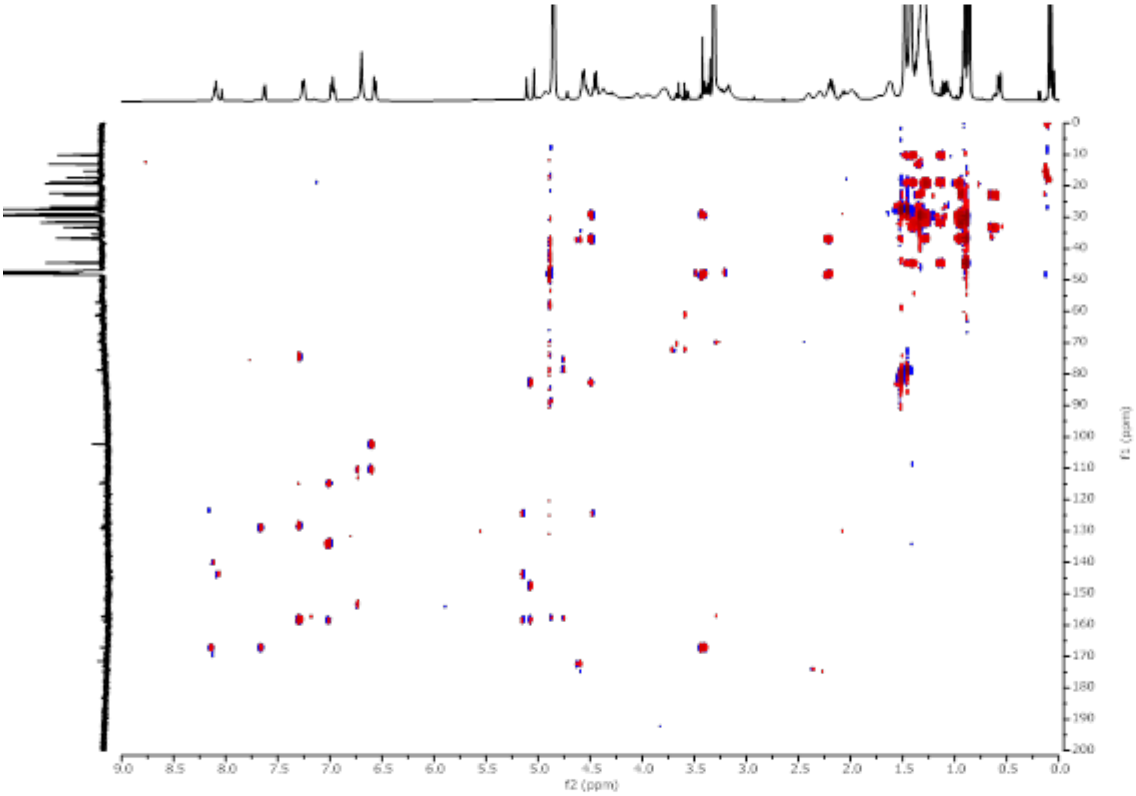

Figure S11. HMBC spectrum of compound 4 in MeOH-d$_4$.

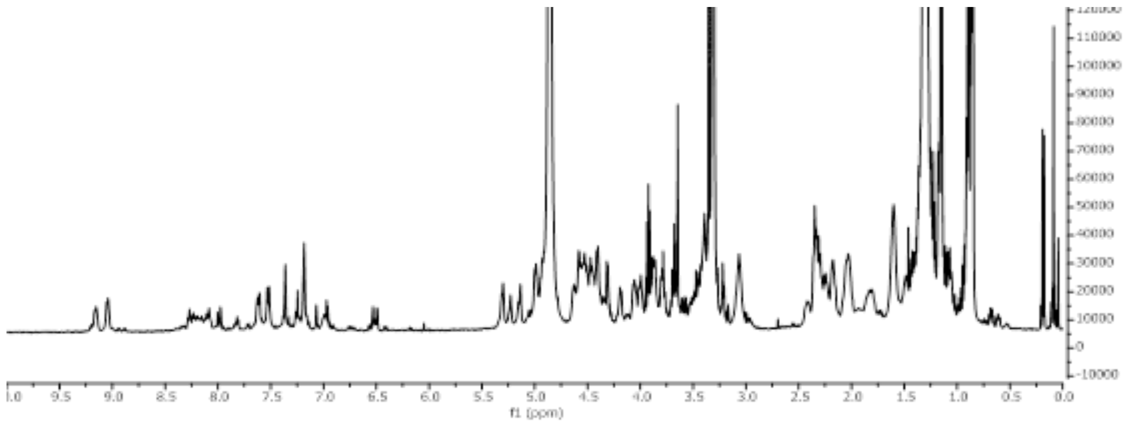

Figure S12. 500 MHz ^{1}H-NMR spectrum of compound 1 in MeOH-d_4.

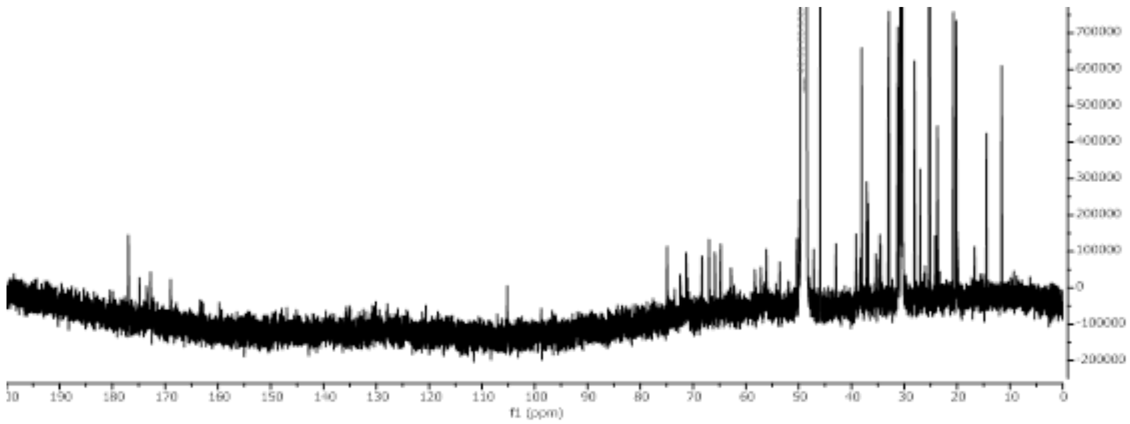

Figure S13. 126 MHz ^{13}C-NMR spectrum of compound 1 in MeOH-d$_4$.

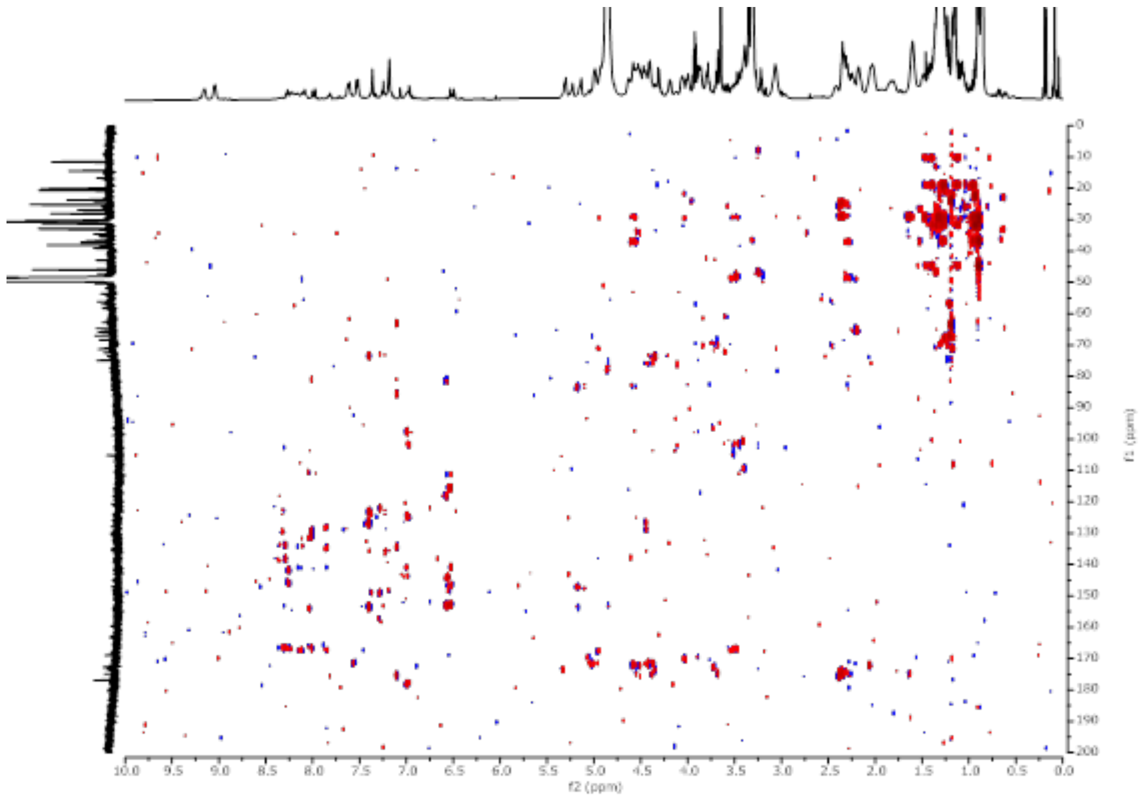

Figure S14. HMBC spectrum of compound 1 in MeOH-d_4.

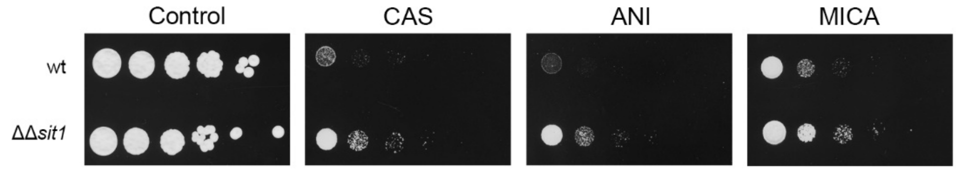

Figure S15. The absence of Sit1 renders *C. albicans* more tolerant to anidulafungin and micafungin. Growth of *C. albicans* wild-type (WT) strain (CAF2-1) and the ΔΔ*sit1* mutant was assessed on SC agar plates (Control) or plates containing 0.75 µg/mL of caspofungin (CAS), anidulafungin (ANI), or micafungin (MICA) after incubation for 4 days at 30 °C.

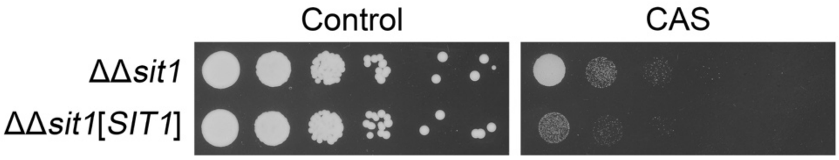

Figure S16. Reintroduction of *SIT1* into the ΔΔ*sit1* mutant strain increases its sensitivity to caspofungin. Growth of the *C. albicans* ΔΔ*sit1* mutant and the ΔΔ*sit1* mutant with *SIT1* reintegrated into the genome (SM183C) was assessed on SC agar plates containing 0.4 µM caspofungin (CAS) and incubated at 30 °C.

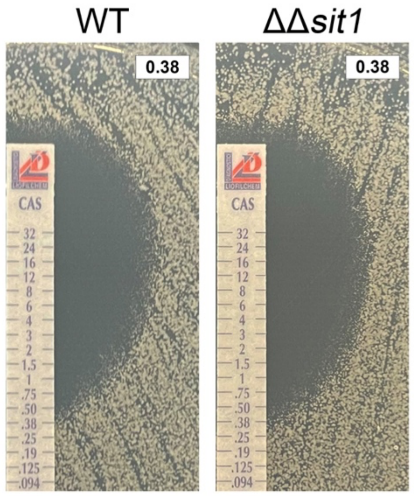

Figure S17. Wild-type (wt, CAF2-1) and ΔΔ*sit1* mutant *C. albicans* strains were tested for susceptibility to caspofungin using E-test strips on SC agar medium, incubated at 30°C for 24 h. Minimum inhibitory concentration (MIC) values (µg/mL) are shown in the corner of each panel.

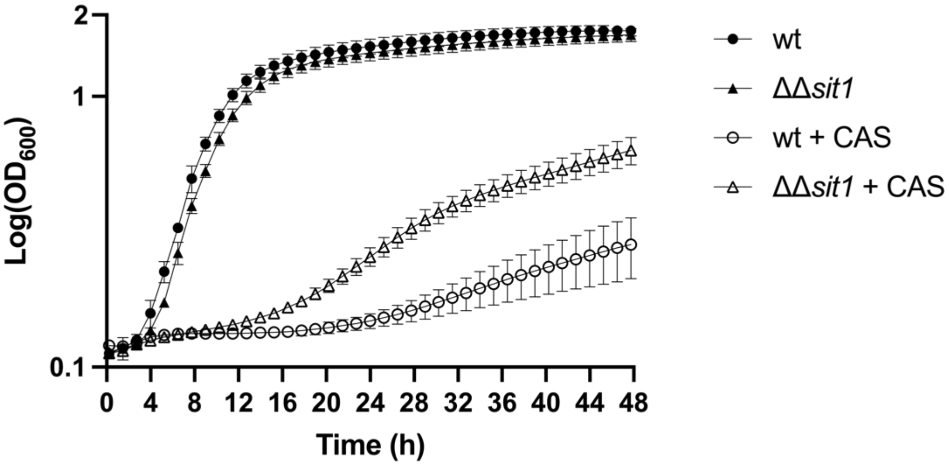

Figure S18. Growth curves of the *C. albicans* ΔΔ*sit1* mutant and wild-type (WT, CAF2-1) strains in the presence of caspofungin. Assays were performed in SC medium using 96-well flat-bottom plates (Nunc Edge). WT and ΔΔ*sit1* cells were grown overnight in SC medium, diluted to an OD_{600} of 0.1, and left untreated or treated with 0.4 μM caspofungin (+CAS). Cell growth was recorded every 30 minutes for 48 hours at 30 °C using a BioTek Epoch2 Microplate Reader (Agilent). Each condition was tested using eight biological replicates.

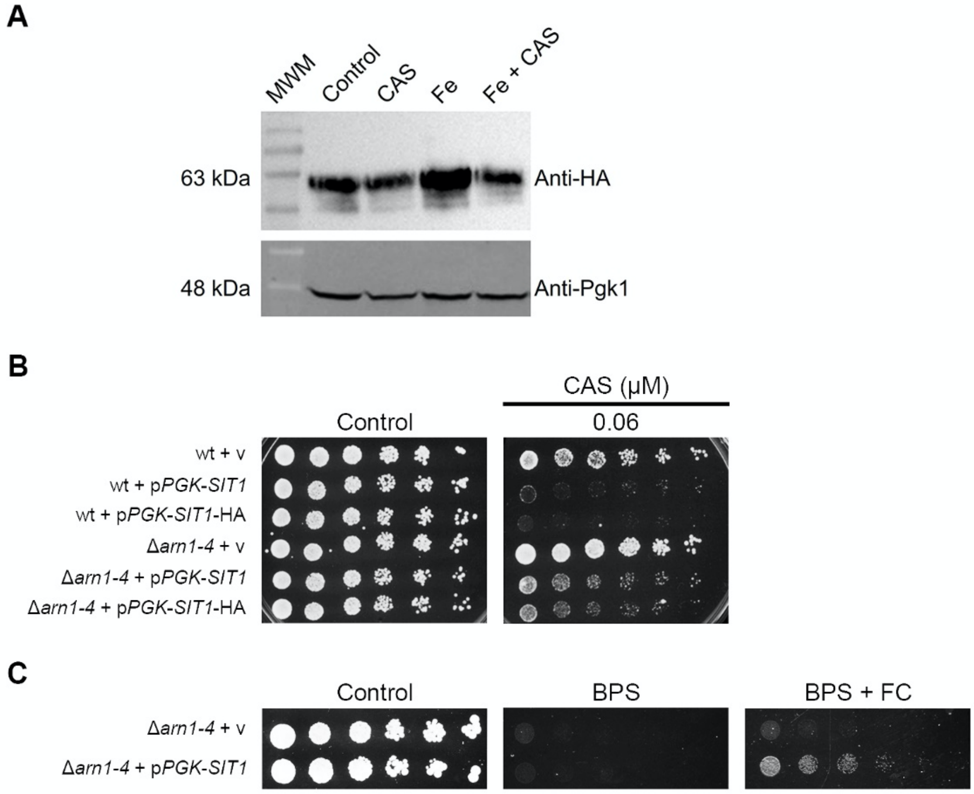

Figure S19. *Saccharomyces cerevisiae* cells transformed with p*PGK-SIT1* express a functional *Ca*Sit1 transporter. (A) Western blot analysis of cells carrying an HA-tagged version of *CaSIT1*. Cells transformed with p*PGK-SIT1*-HA were left untreated (Control) or treated with iron (Fe, 5 mM), caspofungin (CAS, 0.375 µg/mL), or both (Fe + CAS) for 2 hours. Pgk1 was used as a loading control. MWM – Molecular Weight Marker (NZYColour Protein Marker II). **(B)** The caspofungin-sensitive phenotype of *S. cerevisiae* cells expressing *Ca*Sit1 is not affected by HA-tagging of the gene. *S. cerevisiae* wild-type (WT, YPH499) and Δ*arn1-4* mutant strains transformed with plasmids carrying either the untagged or HA-tagged version of Ca*SIT1* were grown on SC-ura agar plates (Control) or plates containing the indicated concentrations of caspofungin (CAS) for 48 hours at 30 °C. **(C)** The *S. cerevisiae* Δ*arn1-4* mutant strain expressing *Ca*Sit1 is capable of resisting iron depletion following ferrichrome (FC) addition. Spot assays were performed on SC-ura plates containing 300 µM BPS (iron-depleted medium), 10 µM ferrichrome (FC), or both (BPS + FC), and growth was recorded after 72 hours of incubation at 30 °C.

References

1. Heymann, P., et al., *The siderophore iron transporter of Candida albicans (Sit1p/Arn1p) mediates uptake of ferrichrome-type siderophores and is required for epithelial invasion.* Infect Immun, 2002. **70**(9): p. 5246-55.
2. Coste, A.T., et al., *Emerging echinocandin-resistant Candida albicans and glabrata in Switzerland.* Infection, 2020. **48**(5): p. 761-766.
3. Yun, C.W., et al., *Desferrioxamine-mediated iron uptake in Saccharomyces cerevisiae. Evidence for two pathways of iron uptake.* J Biol Chem, 2000. **275**(14): p. 10709-15.
